# Global Corrections to Reference Irradiance Spectra for Non-Clear-Sky Conditions

**DOI:** 10.1021/acs.est.2c07359

**Published:** 2023-02-03

**Authors:** Sarah B. Partanen, Kristopher McNeill

**Affiliations:** Institute of Biogeochemistry and Pollutant Dynamics (IBP), Department of Environmental Systems Science, ETH Zurich, 8092Zurich, Switzerland

**Keywords:** incident irradiance, correction factors, satellite data, photochemical processes, non-clear-sky conditions, reference spectra

## Abstract

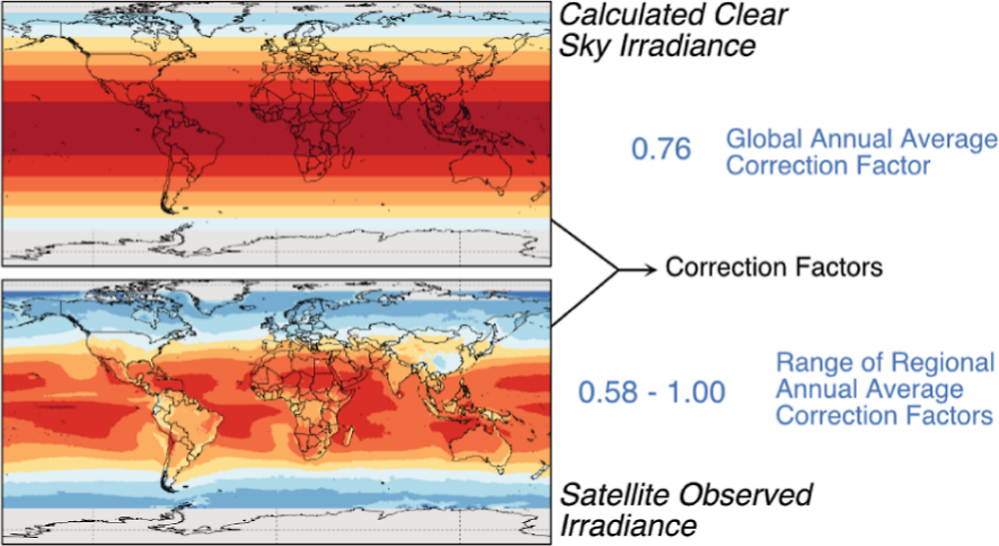

Photochemical reactions in surface waters play important roles in element cycling and in the removal of organic contaminants, among other processes. A central environmental variable affecting photochemical processes in surface waters is the incoming solar irradiance, as this initiates these processes. However, clear-sky incident irradiance spectra are often used when evaluating the fate of aquatic contaminants, leading to an overestimation of contaminant decay rates due to photochemical transformation. In this work, incident irradiance satellite data were used to develop global-scale non-clear-sky correction factors for commonly used reference irradiance spectra. Non-clear-sky conditions can decrease incident irradiance by over 90% depending on the geographic location and time of the year, with latitudes above 40°N being most heavily affected by seasons. The impact of non-clear-sky conditions on contaminant half-lives was illustrated in a case study of triclosan in lake Greifensee, which showed a 39% increase in the triclosan half-life over the course of a year under non-clear-sky conditions. A global annual average correction factor of 0.76 was determined as an approximate way to account for non-clear-sky conditions. The correction factors are developed at monthly and seasonal resolutions for every location on the globe between 70°N and 60°S at a 4 km spatial resolution and can be used by researchers, practitioners, and regulators who need improved estimates of incident irradiance.

## Abstract 2

Incident irradiance is a key variable affecting photochemical processes, but an assumption of clear sky conditions is often made. This work provides global-scale correction factors for researchers and practitioners who need more realistic estimates of incident irradiance.

## Introduction

Incident irradiance, or the light from the sun which reaches the surface of the earth, is the central variable affecting photochemical processes in the environment. Having reliable estimates of irradiance values is important for accurate predictions of the rates of photochemical processes. For example, since photochemical transformation can be an important removal process for some organic contaminants in environmental systems,^1−4^ accurate irradiance values are needed to properly predict photochemical rates. Using a laboratory light source to approximate environmental solar irradiance often represents a best-case scenario of summer noon (or even more intense) sunlight^1,5,6^ and can be non-representative of realistic environmental conditions over the course of a year.

Besides the spectra from laboratory light sources, there exist good clear-sky reference irradiance spectra from tools such as the Simple Model of the Atmospheric Radiative Transfer of Sunshine (SMARTS).^8−22^ These irradiance spectra are more representative of environmental conditions as they account for factors such as seasonality, position on earth, and aerosol loading. However, cloud cover is the largest factor that decreases incident irradiance from reference clear sky values, and this factor is not accounted for in the reference spectra.^10^ The magnitude of the effect of cloud cover on incident irradiance and its variability by season and position on earth are not well understood in the context of photochemical modeling, although some progress is being made in this area.^11^

In the present work, we sought to better understand the effect of factors that decrease incident irradiance from clear sky levels by comparing satellite-derived surface irradiance values to clear sky values. Satellite data are promising for this purpose, as they are collected on a massive scale by many organizations including NASA and the European Space Agency and are often available for researchers to use free of charge.^12,13^ Satellite-based irradiance data can also better reflect the “true” irradiance values, as the data processing algorithms account for factors such as aerosol loading and cloud cover.^14,15^ In this work, we have used satellite-based measurements and calculated reference irradiance spectra to generate a set of correction factors (CFs) that can be used on a variety of spatial and temporal scales to give a more realistic estimate of incident irradiance. We anticipate that the CFs generated in this work will be useful for researchers who wish to better predict realistic environmental fates of chemical contaminants due to photochemical processes or other transformation processes that rely on the sun’s energy.

## Methods

### Terminology

Three different forms of ground-level photosynthetically active radiation (PAR) irradiance values were used in this work:

1. Indirectly measured real-sky PAR; from satellite data
2. Modeled clear-sky PAR; from the SMARTS tool
3. Directly measured real-sky PAR; from monitoring stations in the United States

These three data sources are discussed in the following sections.

The CFs or ratios generated in this work relate indirectly measured real-sky PAR to clear-sky PAR as shown in eq 1.
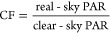
1

### Data Sources

#### Real-Sky Incident Irradiance

The Moderate Resolution Imaging Spectroradiometer (MODIS) is an instrument on board the Terra and Aqua NASA satellites which acquires data in 36 spectral bands over the entire surface of the Earth every 1–2 days. Raw data from the satellites is processed, archived, and distributed by a series of Distributed Active Archive Centers (DAACs). Incident PAR data from the Land Processes (LP) DAAC and the Ocean Biology (OB) DAAC were used as indirect measurements of ground-level real-sky PAR in this work.

Land surface PAR (MCD18A2 Version 061) is a combined MODIS Terra and Aqua product from the LP DAAC consisting of gridded level 3 PAR data at 1 km spatial resolution and 3 h temporal resolution in units of energy per unit area (W m^–2^).^16^ NASA data levels refer to the level of processing that raw data (level 0) have undergone, where variables in level 3 data have been mapped to a uniform space-time grid scale.^17^ This PAR data product exists over land surfaces (including over lakes and rivers) only; there is no data over ocean surfaces. The PAR values are developed from MODIS data based on a look-up-table (LUT) approach where surface reflectance data under clear-sky conditions is first determined, and then, incident PAR is calculated from the surface reflectance and top-of-atmosphere radiance/reflectance by searching the LUTs. The LUTs are precalculated from simulations of atmospheric radiative transfer models that represent a comprehensive range of atmospheric conditions (aerosol or cloud optical depths).^15^

Daily average PAR at the ocean surface is generated by NASA’s OB processing group at 4 km spatial resolution in quantum PAR units (mol photon m^–2^ day^–1^). Monthly average PAR data is available as a level 3 product.^18^ Ocean surface PAR is modeled using plane-parallel theory under the assumption that the effects of clouds and the clear atmosphere can be decoupled. This allows for the atmosphere to be modeled as a clear-sky atmosphere positioned above a cloud layer.^14^ A combined MODIS-Terra and -Aqua product is not available for ocean surface PAR as it is for land surface PAR. However, Frouin et al. compared monthly PAR from MODIS-Terra to merged data from three sensors (MODIS-Terra, MODIS-Aqua, and SeaWiFS) and concluded that the two data sets were very similar with no discernible biases.^19^ Therefore, monthly ocean surface PAR data from MODIS-Terra was used here.

#### Clear-Sky Incident Irradiance

Clear-sky PAR values were modeled in this work using SMARTS.^20−22^ Briefly, global horizontal PAR irradiance (400–700 nm) in units of W m^–2^ was modeled on an hourly basis for a full year between 70°N and 60°S in 5° increments. A full list of the input parameters used in the SMARTS tool can be found in the Supporting Information (Tables S1 and S2), as can a discussion of the impact of the reference atmosphere as an input parameter on the SMARTS model output (Figure S1).

#### Validation Incident Irradiance

PAR data from the US National Oceanic and Atmospheric Administration’s (NOAA) Surface Radiation Budget (SURFRAD) network of measurement stations was used for validation of the CFs developed in this work.^23^ Monthly average radiation data were downloaded from https://gml.noaa.gov/aftp/data/radiation/surfrad/averages/ (last accessed July 10, 2022) for seven sites representing diverse climates across the United States (Table S4).

### Data Processing and Analysis

**Figure 1:**
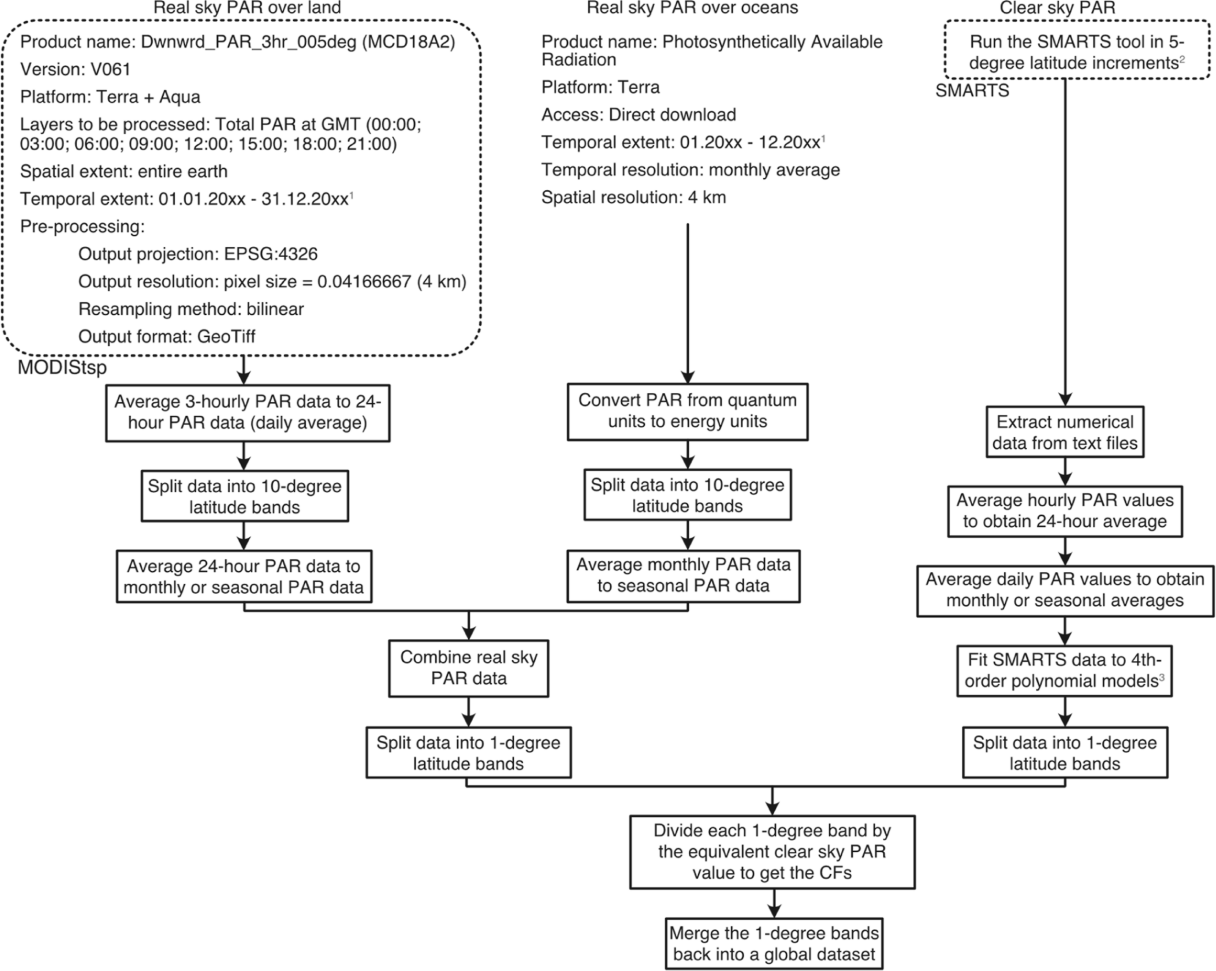
Summary of the data processing steps completed in this work. (1) 2004–2016, 2019, and 2020. (2) See Figures S2 and S3. (3) See Table S3 and Figures S4 and S5.

A visual summary of the data processing workflow can be seen in Figure 1. The steps shown within dashed line boxes were performed using the specific model or R package noted in the figure, while the remainder of steps were completed within RStudio (Version 2022.07.2). For a full list of the R packages used in this work, see Table S5. The output of the data processing and modeling steps is the generation of CFs at a spatial resolution of 4 km from 60°S to 70°N and on both monthly and seasonal timescales. Data from the following years were used in this work: 2004–2016, 2019, and 2020. Data from 2017 to 2018 were excluded from this analysis because one of the data sources was being updated to a new version at the time of the analysis, and data from these years were unavailable to download.

Indirectly measured real-sky PAR data over land were downloaded and pre-processed using the MODIStsp package in RStudio.^24^ Details of the pre-processing parameters are shown in Figure 1, but briefly, the product “Dwnwrd_PAR_3h_005deg (MCD18A2)” version 6.1 was downloaded from both MODIS-Terra and -Aqua platforms. The output projection of the data was changed from the native NASA sinusoidal projection (EPSG:4008) to match that of the indirectly measured real-sky PAR data over oceans (EPSG:4326), and the resolution of the output was aggregated from 1 km to 4 km spatial resolution using a bilinear resampling method, also to match the spatial resolution of the real-sky PAR data over oceans. All tiles over the surface of the earth were selected. Once the 3 h data was downloaded and pre-processed using MODIStsp, the data was further processed in RStudio using the Terra package.^25^ In brief, each pixel of the 3 h data was averaged first into daily values and then either monthly or seasonal values.

Indirectly measured monthly real-sky PAR data over oceans was accessed directly from the OB DAAC (https://oceandata.sci.gsfc.nasa.gov/directdataaccess/Level-3%20Mapped/Terra-MODIS, last accessed October 7, 2022).^18^ The data is available in units of mol photon m^–2^ day^–1^ (quantum PAR) and thus needed to be converted to W m^–2^ (energy PAR) to match the units of the real-sky PAR data over land. The determination of the conversion factor between quantum PAR and energy PAR is discussed in the Supporting Information. The real-sky PAR data was combined into a single data set using the Terra package in R. A comparison between the real-sky PAR data sets over land and ocean can be found in the Supporting Information (Tables S6 and S7 and Figure S6).

Clear-sky PAR (monthly average global horizontal PAR irradiance) modeled using the SMARTS tool has low interannual variability (99.7% of values within 2% of each other for 2019 and 2018 values); therefore, modeled data from 2019 were used for all subsequent analyses, except in the case of leap years (2004, 2008, 2012, 2016, and 2020 in the range of years studied here), in which case data from 2020 were used. The clear-sky PAR was modeled in 5° increments; however, to calculate the CFs, clear-sky data in 1° increments were required. Clear-sky PAR for a given month across latitudes from 60°S to 70°N was therefore fit to a series of fourth-order polynomial equations to obtain values at latitudes in between those directly modeled using the SMARTS tool. The fitting parameters and plots of the data fit to polynomial models can be seen in Table S3 and Figures S4 and S5.

### Equations Governing Direct Photolysis

The impact of non-clear-sky conditions on the half-life of triclosan in lake Greifensee was used as a case study in this work. The following equations were used to model triclosan behavior in the lake epilimnion.

The rate constant for direct photodegradation of triclosan, *k*_TCS_^dir^ (s^–1^), in an aquatic system can be expressed according to eq 2
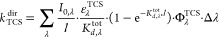
2where *I*_0,λ_ is the incident irradiance (mmol photons cm^–2^ s^–1^ nm^–1^),  is the pathlength of light through water (cm), ε_λ_^TCS^ is the molar absorptivity of triclosan (M^–1^ cm^–1^), *K*_*d*,λ_^tot^ is the diffuse attenuation coefficient of the waterbody (cm^–1^), and Φ_λ_^TCS^ is the direct photolysis quantum yield of triclosan (mol triclosan mol photon^–1^).

Note that in this formulation of eq 2, all parameters are wavelength-dependent, represented by a subscript λ. The first three terms in eq 2 represent the rate of light absorbance of the chromophore of interest (in this case triclosan). The factor  is the total fraction of light absorbed by all chromophores in the system (i.e., triclosan and DOM), and the factor  represents the fraction of light absorbed by triclosan. In this work, we assumed that the contribution of triclosan to *K*_*d*,λ_^tot^ is small compared to the contribution of dissolved organic matter (DOM); therefore, *K*_*d*,λ_^tot^ could be modeled as a function of dissolved organic carbon according to Morris et al.^26^

Triclosan has an environmentally relevant p*K*_a_ value (8.05 ± 0.03)^27^ and is therefore present in the environment in two different forms: the phenolate form and the phenol form. In lake Greifensee, triclosan is mainly present in its phenolate form based on a measured lake pH of 8.6.^28^ Additionally, it is the phenolate form that is most photolabile due to the larger overlap of the phenolate absorbance spectrum with the solar spectrum. We therefore assume that *k*_TCS_^dir^ modeled in eq 2 is primarily due to the reaction of the phenolate form. The apparent *k*_TCS_^dir^ for triclosan can subsequently be approximated by multiplying *k*_TCS_^dir^ of the phenolate form by the fraction of triclosan that is in the phenolate form, according to eq 3.
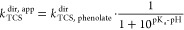
3Finally, the direct photolysis half-life of triclosan is calculated according to eq 4
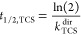
4

## Results and Discussion

### Global CFs

**Figure 2:**
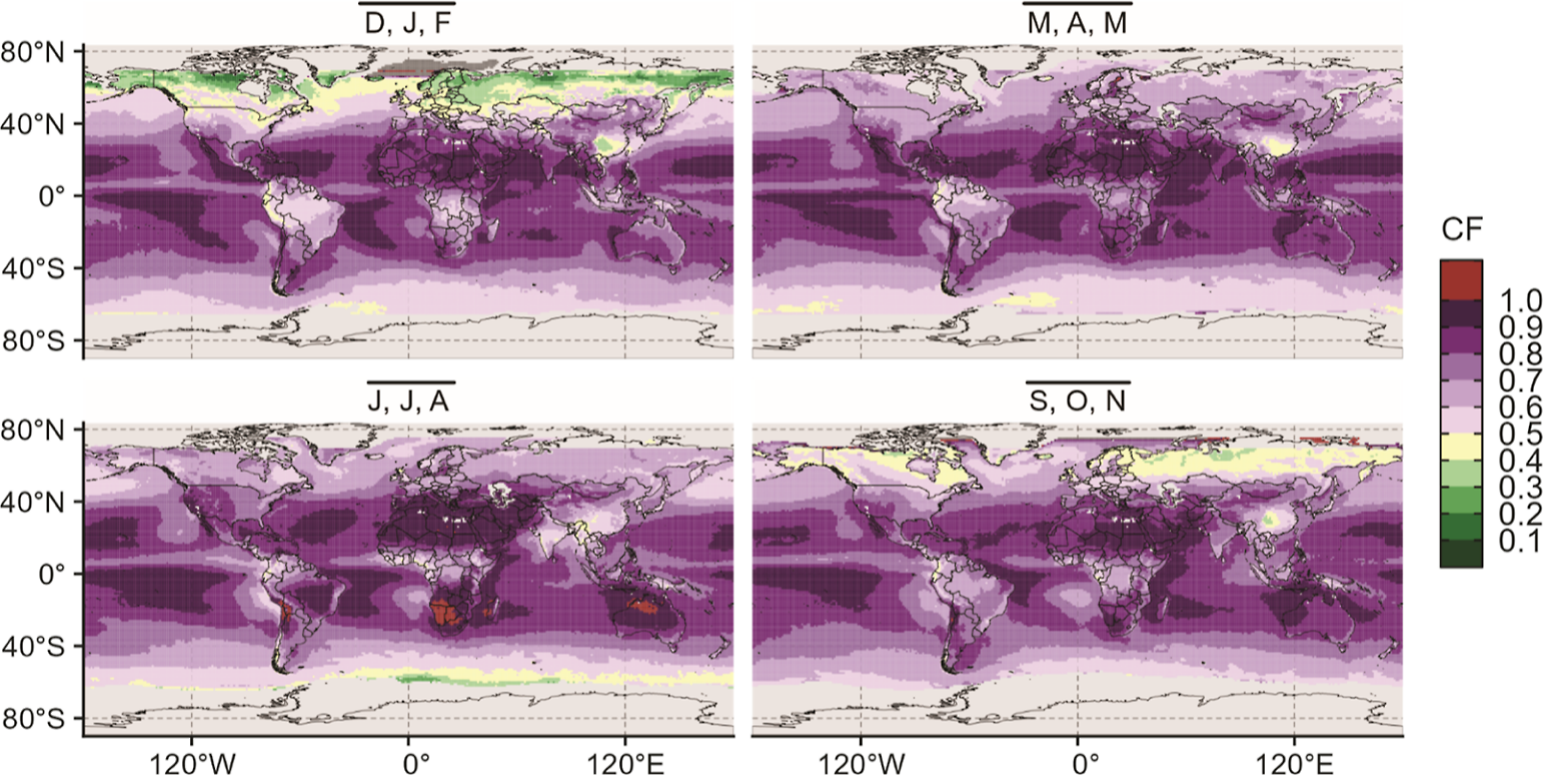
Global maps of CFs shown as average seasonal values.  = the average of December, January, and February;  = the average of March, April, and May;  = the average of June, July, and August; and  = the average of September, October, and November.

We generated CFs to account for non-clear-sky conditions for every location on the globe between 70°N and 60°S at a 4 km spatial resolution and on seasonal and monthly timescales (Figures 2 and S7–S10). The global annual average CF is 0.76, and the 90% confidence interval of global annual average CFs is 0.54–0.92. Seasonal and monthly 90% confidence intervals are shown in Tables S8 and S9, respectively. Seasonal CFs at 1° spatial resolution are provided as separate files within the Supporting Information (.xlsx and .tif).

### Variability of CFs

**Figure 3:**
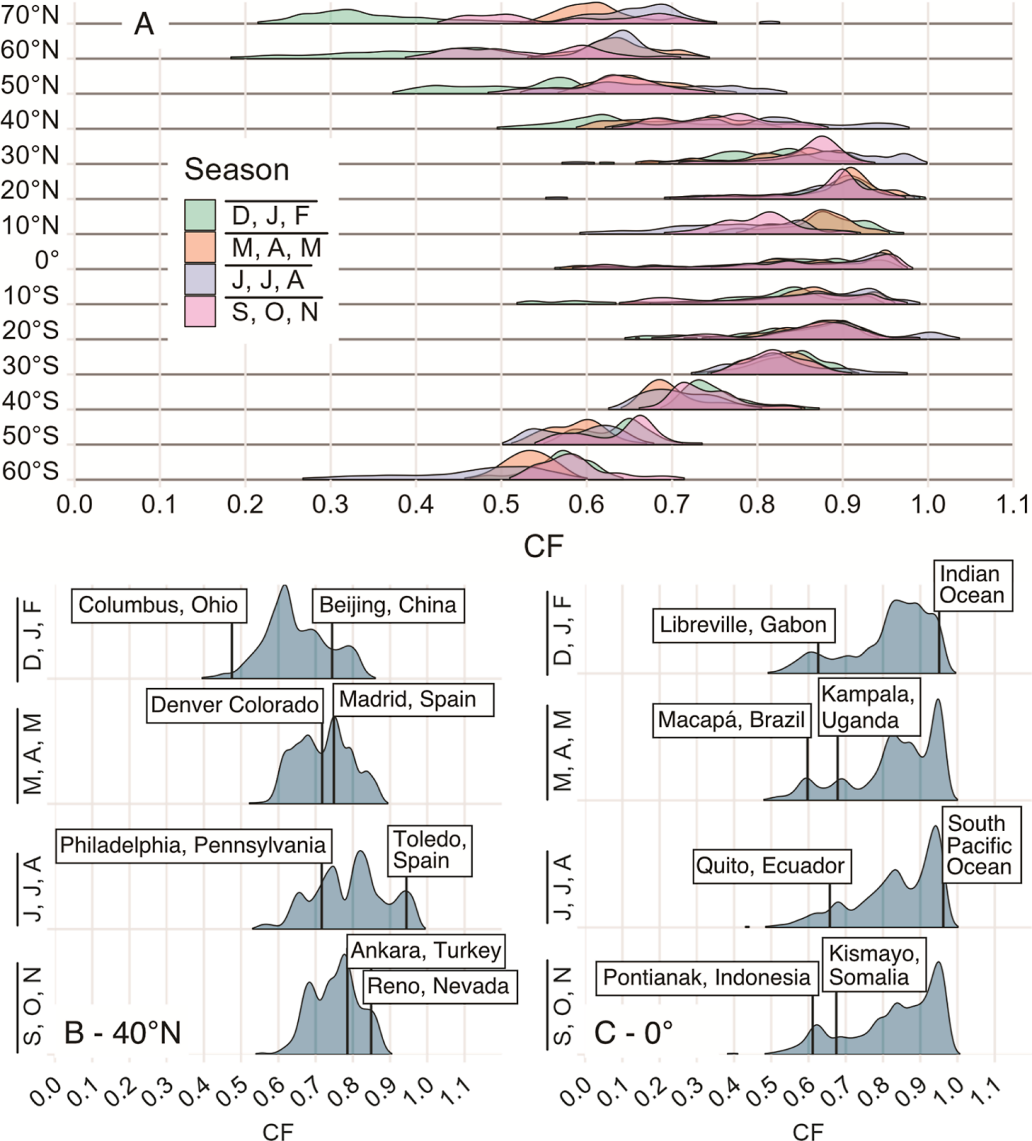
(A) Ridgeline plots of CFs as a factor of the latitude and season. (B) Ridgeline plot of CFs at 40°N highlighting the seasonal variability of the ratios. CFs for illustrative locations are labeled. (C) Ridgeline plot of CFs at the equator highlighting the seasonal variability of the ratios. CFs for illustrative locations are labeled.

Variability in the CFs can be examined from both a spatial and a temporal standpoint. Figure 3A shows the spatial variability in the CFs at latitude bands in 10° increments from 60°S to 70°N and the seasonality of the CFs. Figure S11 displays the same data broken out by the latitude band to better highlight the seasonal variability of the CFs. Although there are clear spatial and seasonal variations in the CFs, the data resist a simple or neat interpretation.

There is a clear shift to CFs closer to 1 as the latitude approaches the equator (0°) from both the northern and southern hemispheres. Notably, however, the equator is not the latitude with the highest CFs (i.e., real-sky irradiance closest to the clear-sky irradiance), likely due to the inter-tropical convergence zone, which appears as a band of clouds near the equator.^29^ Instead, the CFs closest to 1 are found closer to 20°S. The impact of non-clear skies on incident irradiance is not symmetrical about the equator, which is not surprising given the differences in land distribution and cloud patterns. Regional differences in cloud cover are caused by a variety of incompletely understood processes including atmospheric circulation^30,31^ and orographic effects,^32^ among others. Additionally, many of the distributions of CFs within a latitude band are very broad; even the tightest distributions spread over ∼30% of the range of possible ratios, indicating that there is significant longitudinal variation in the CF. This indicates that a single value for the entire latitude band would not be meaningful.

The seasonal trends in the CFs at different latitude bands are quite variable. For many latitudes, there is a high degree of overlap in the distributions of values for each season, indicating low inter-seasonal variability (for example, Figure 3B,C). In contrast, northern hemisphere latitudes above 40°N are strongly affected by the season, as evidenced by the shift of CFs to lower values and the increase in the spread of the distributions of ratios. For example, the interquartile range (the spread of the middle 50% of values) of CFs for  at 50°N is 0.12, whereas at 50°S, it is 0.06 (see Figure S11 in the Supporting Information).

Variability in the CF was also examined by global region, as defined in Table S10 in the Supporting Information. This way of summarizing the data lends itself to use in modeling efforts or regulatory registration of chemicals. Seasonal and annual average CFs for each subregion are shown in Table 1, and the distributions of the data for each subregion can be seen in Figures S12–S16. Note that the CFs in Table 1 represent the mode of the distribution, not the mean, as many of the distributions are not normally distributed. In the absence of measurements or more sophisticated models, we believe that the mode of the CF distribution for each subregion is a reasonable ratio by which to correct clear-sky reference incident irradiance spectra, particularly for long-lived processes (half-life on the timescale of months or years).

**Table 1:** Representative Seasonal and Annual CFs for Global Subregions^a^

		subregion				
region	season	East Africa	Mid-Africa	North Africa	South Africa	West Africa
Africa	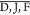	0.63	0.61	0.89	0.76	0.88
	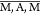	0.77	0.70	0.90	0.87	0.91
	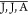	0.98	0.69	0.98	1.00	0.92
	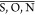	0.76	0.62	0.95	0.91	0.88
	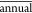	0.73	0.69	0.95	0.99	0.88

		subregion			
region	season	Caribbean	Central America	North America	South America
Americas	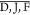	0.72	0.84	0.49	0.55
	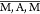	0.74	0.91	0.67	0.75
	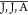	0.79	0.82	0.67	0.72
	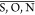	0.70	0.86	0.52	0.77
	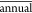	0.73	0.85	0.67	0.73

		subregion				
region	season	Central Asia	East Asia	South Asia	Southeast Asia	West Asia
Asia	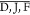	0.51	0.75	0.87	0.60	0.91
	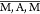	0.77	0.77	0.90	0.68	0.90
	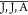	0.84	0.67	0.98	0.70	0.97
	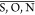	0.73	0.79	0.85	0.62	0.94
	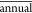	0.77	0.77	0.85	0.62	0.93

		subregion			
region	season	East Europe	North Europe	South Europe	West Europe
Europe	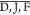	0.37	0.43	0.58	0.44
	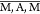	0.63	0.64	0.67	0.63
	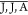	0.66	0.63	0.90	0.64
	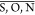	0.55	0.48	0.72	0.52
	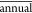	0.64	0.63	0.72	0.63

		subregion	
region	season	Australia and New Zealand	Melanesia
Oceania	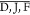	0.82	0.63
	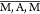	0.93	0.66
	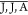	0.99	0.60
	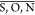	0.89	0.64
	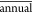	0.84	0.65

a The CFs represent the mode of the distribution of values for that subregion and time period.

**Figure 4:**
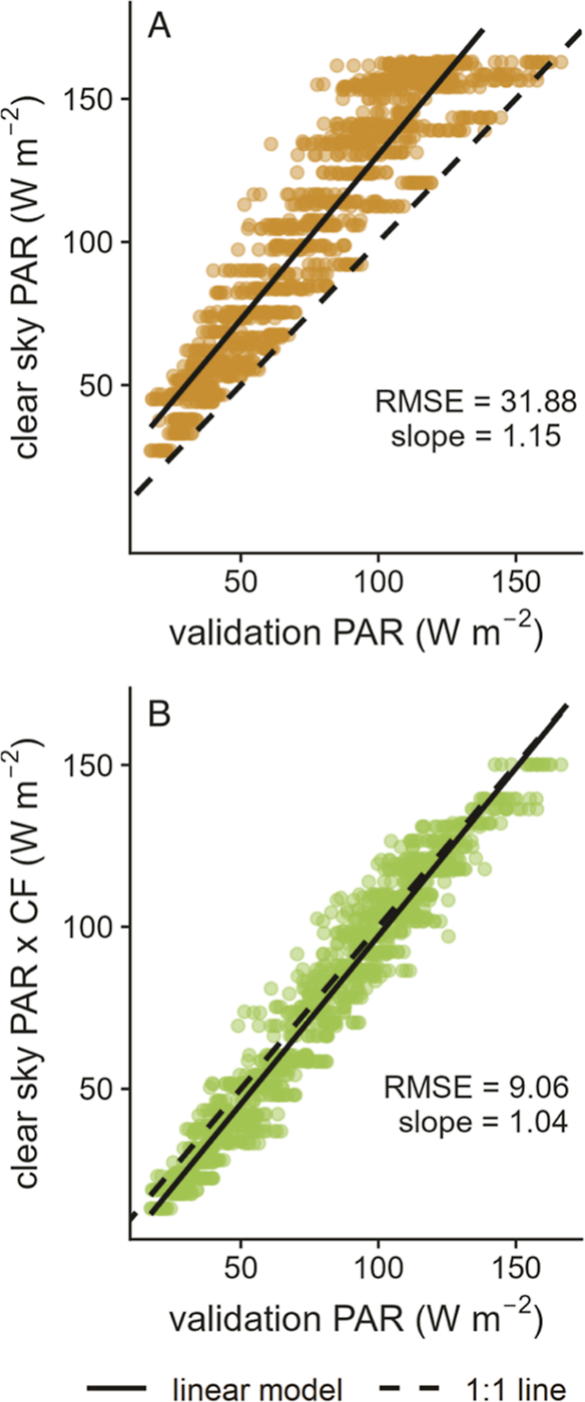
Comparison of directly measured real-sky PAR data to (A) clear-sky PAR modeled using the SMARTS tool for 2020 and (B) PAR values estimated by multiplying clear-sky PAR by the CFs determined in this work.

Inter-annual variability in the CF was explored by mapping the standard deviation of the CFs for 15 years of satellite data, shown in Figure S17. The standard deviation in CFs over much of the Earth’s surface is between 0.01 and 0.1 CF units for all months of the year. This means that for a given month and location on the earth’s surface, the CF has not varied by more than 10% over the last 15 years. The low inter-annual variability of the CFs can also be seen from the validation of the ratios against directly measured real-sky PAR discussed below (Figure 4). Based on these analyses, we feel comfortable providing mean values of 15 years of CFs; this is what is presented in Figure 2.

An important finding from this work is that clear-sky reference irradiance spectra are very rarely an accurate reflection of real-world conditions. The consequences of overestimating the incident irradiance can vary. For example, for some subregions, especially during June, July, and August, the CFs are within 10% of the clear-sky PAR values. For these regions, clear-sky reference incident irradiance spectra could be used in photochemical models without introducing large errors. In contrast, some regions in the northern hemisphere only receive 50% of the clear sky irradiance during parts of the year, which has strong implications for pollutant lifetimes. The effect of accounting for non-clear-sky conditions on pollutant lifetimes is explored in the case study discussed below.

### Validation

The CFs developed in this work were validated using directly measured real-sky PAR data from the US NOAA SURFRAD network of measurement stations.^23^ Monthly average PAR data were retrieved for the same 15 years as were used to develop the CFs (see section “Data Processing and Analysis” mentioned above). These data were compared to both monthly average clear-sky PAR data modeled using the SMARTS tool at the station locations during 2020 and the monthly average clear-sky PAR data multiplied by the CF (i.e., clear-sky PAR data corrected for non-clear-sky conditions) for a given station location (Figure 4). Therefore, the points in Figure 4 represent monthly average real-sky PAR directly measured by the SURFRAD stations regressed against either the monthly average modeled clear-sky PAR (Figure 4a) or the same monthly average clear-sky PAR multiplied by the CFs generated in this work (Figure 4b).

Unsurprisingly, clear-sky PAR modeled using the SMARTS tool overestimates the directly measured real-sky PAR irradiance at the SURFRAD stations (Figure 4A). When the CFs generated in this work are multiplied by the clear-sky PAR values, that is, when clear-sky PAR data are corrected for non-clear-sky conditions, there is very good agreement with the directly measured real-sky PAR values (Figure 4B). Note that the mean values of 15 years of CFs were used in the validation and that even though an average CF is applied, the PAR values estimated by multiplying clear-sky PAR by the CFs still match the directly measured real-sky PAR in a single year extremely well.

### Case Study: Triclosan in Lake Greifensee

**Figure 5:**
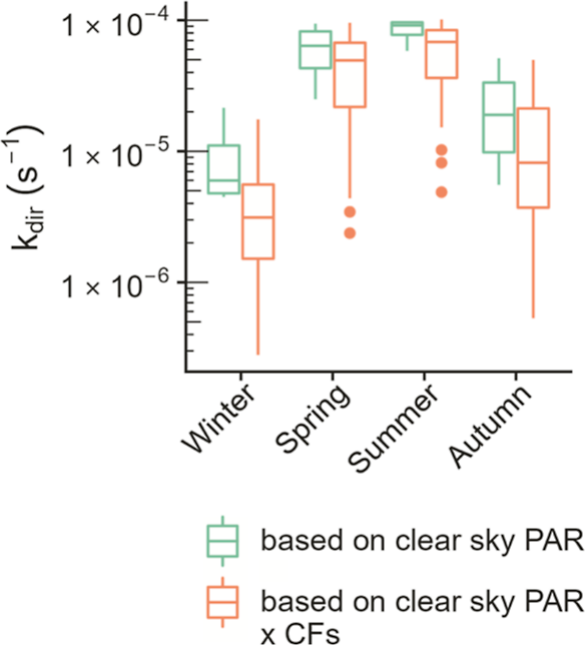
Range of seasonal direct photolysis rate constants of triclosan at the surface of lake Greifensee. Green values were calculated using clear-sky PAR, and orange values were calculated using clear-sky PAR multiplied by the CFs generated in this work. Note that the *y*-axis is log-scale. The lower and upper edges of the boxplots represent the first and third quartiles, the middle line represents the median, the ends of the whiskers represent the largest or smallest values no further than 1.5 × the interquartile range, and the circle symbols represent outliers.

Lake Greifensee is a small eutrophic lake in Switzerland. The phototransformation of triclosan in lake Greifensee was examined in 2002 by Tixier et al.^28^ who found that triclosan is susceptible to direct photodegradation and is insensitive to indirect photochemical processes. Here, the direct photolysis rate constant and half-life of triclosan in lake Greifensee were calculated using eq 2 under clear-sky conditions and accounting for non-clear-sky conditions in 2020 (see Figure S18 for wavelength-dependent input parameters to eq 2). Clear-sky conditions were simulated by using a reference incident irradiance spectrum modeled using the SMARTS tool at the latitude and longitude of lake Greifensee (47.35°N, 8.68°E) from 300 to 700 nm (Figure S18C). Non-clear-sky conditions were accounted for by multiplying the clear-sky reference spectrum by the CFs at lake Greifensee estimated in this work for each day of 2020. Box plots of direct photolysis rate constants at the surface of lake Greifensee for both clear-sky and accounting for non-clear-sky conditions are presented in Figure 5.

The spread of the direct photolysis rate constants for triclosan in Lake Greifensee is wider when non-clear-sky conditions are considered (Figure 5). This makes sense, as a wider range of potential incident irradiance values would directly lead to a wider range of observed direct photolysis rate constants. In addition, the mean direct photolysis rate constant is significantly lower for all seasons under real-sky conditions than under clear-sky conditions (Table S11).

An average half-life for triclosan in lake Greifensee was also calculated for 2020. The calculation was done by first determining an average annual rate constant for direct photolysis, accounting for the fact that lake Greifensee is holomictic and experiences regular mixing during the months of December to March. Thus, the direct photolysis rate constants must be calculated using a different lake depth depending on the month. The mean depth of lake Greifensee is 17.8 m,^33^ so this was assumed to be the lake depth during the months of December to March. We assumed that the lake is stratified during the other months of the year with an epilimnion depth of 5 m.^28^ Under these depth conditions and accounting for non-clear-sky conditions, the average rate constant for the direct photolysis of triclosan in lake Greifensee in 2020 is 5.48 × 10^–7^ s^–1^, and the average half-life is 14.6 days or 39% longer lived than that under clear-sky conditions.

### Comparison to Other Data Sources and Tools from Other Fields

Research fields beyond environmental photochemistry also benefit from accurate surface level values of incident irradiance. In particular, the photovoltaic industry requires very accurate forecasts of solar irradiance, as abrupt variations in irradiance have the potential to degrade the quality of the photovoltaic power.^34^ Silicon-based solar cells are the most popular type of solar cell currently on the market and have a useable wavelength range of approximately 300–1100 nm. This band gap means that researchers in the photovoltaic industry primarily use downward shortwave radiation (DSR) as a parameter in their incident irradiance models. The use of DSR coupled with the fundamentally different requirements of the photovoltaic industry (forecasting rather than static factors, very fine-grained spatial and temporal resolution) means that irradiance models developed for use in the photovoltaic industry are not generally appropriate for use in photochemical models. Furthermore, knowing surface incident irradiance is not the same as having a CF for non-clear-sky conditions. It is the comparison to the reference clear-sky irradiance values that allows for the practical use of the incident irradiance data.

### Assumptions and Limitations

It is important to note the assumptions that the CFs generated in this work rest upon so that they are not applied out of context. First, we are placing some trust in the veracity of the MODIS satellite products generated by the OB- and LP-DAACs. The theoretical basis of the algorithms used to process the raw satellite data into ocean and land PAR products is well documented and well validated.^14,15^ However, as with any satellite product, there are limitations. For example, the ocean PAR algorithm ignores diurnal variability of cloud cover, as it is a 24 h-averaged product, and the land PAR algorithm can result in uncertainties under complex cloud cover due to the LUT-based approach. In this work, we are using monthly or seasonal averages of the data, meaning that we are ignoring variability on finer temporal scales than by month or by season, respectively. We believe that the level of data aggregation in this work is appropriate given the intended use of the CFs in calculating pollutant fate or similar calculations over longer timescales. We are also ignoring variability in incident irradiance on a spatial scale finer than approximately 4 km. Although there is certainly variability in cloud cover over scales smaller than 4 km, in this work, we are more interested in providing regional- and global-scale coverage of CFs. Similar to the temporal aggregation, we believe that the applied level of spatial aggregation will best serve researchers and practitioners who wish to use these CFs.

In this work, we have also made assumptions about the generalizability of PAR irradiance to the entire solar spectrum and the applicability of the CFs to all wavelengths of the reference spectra. Specifically, we are assuming that clouds are the main factor impacting ground-level irradiance.^10,35,36^ Since cloud droplets are larger than the wavelengths of light we are considering, light is scattered according to Mie theory. Mie scattering affects all wavelengths of light in the visible spectrum more or less equally; thus, the process can be considered wavelength-independent.^37^ Therefore, we feel comfortable applying the CFs developed in this work across the entire reference solar spectrum.

### Implications for Photochemical Modeling

Translating work done in the laboratory into knowledge of what is occurring in the environment is not always straightforward. This work attempts to make this translation easier for one important parameter in photochemical modeling—the incident irradiance. CFs for reference incident irradiance spectra were generated on a 4 km spatial scale and on monthly and seasonal temporal scales. These CFs are not only valuable to researchers modeling pollutant fate in the environment but also to regulators wanting to estimate non-idealized contaminant lifetimes in aquatic systems.

The case study on triclosan in lake Greifensee presented in this work highlights the fact that the impact of accounting for non-clear-sky conditions when calculating pollutant half-lives is not fully encompassed by a single average value. The annual average half-life of triclosan in lake Greifensee increases by about 39% when non-clear-sky conditions are considered, but in addition, there is generally more variability in the direct photolysis rate constants that would be observed in nature. The wide range of direct photolysis rate constants modeled here indicates that reporting a single rate constant value for a given contaminant is so location- and time-of-year-dependent as to be completely parochial and non-generalizable. Reporting ranges of expected pollutant rate constants or half-lives gives a much more realistic picture of the true environmental behavior of aquatic contaminants.

It should also be noted that while the case study presented here focuses on triclosan, which primarily transforms via direct photolysis in the environment, the CFs developed in this work can also be applied when modeling indirect photochemical processes. For example, the rate of formation of reactive oxygen species (e.g., singlet oxygen) in the environment is highly dependent on the amount of incoming solar radiation,^38^ so incorporating the CFs into these types of modeling efforts would have implications for the calculated steady-state concentration of singlet oxygen and ultimately the half-lives of pollutants reacting with singlet oxygen.

The impact of accounting for non-clear-sky conditions on pollutant half-lives in aquatic systems depends on the location on earth, time of the year, and the user. For some locations, using reference irradiance spectra that assume clear-sky conditions will introduce minimal error. For some users, even if accounting for non-clear-sky conditions results in a factor of 2 difference in the pollutant half-life, this error may be acceptable for their use. In other cases, the impact of non-clear-sky irradiance on parameters like the pollutant half-life will be significant. For example, we can consider the fate of triclosan in Moberly Lake, a small lake in northern British Columbia, Canada, at a higher latitude than lake Greifensee; the average triclosan half-life in 2020 was modeled to increase from 120 to 200 days when non-clear-sky conditions were considered, an increase of 66% (see Figure S19 for the range of seasonal triclosan direct photolysis rate constants in Moberly Lake).

Until now, it has been difficult to accurately assess the magnitude and scope of the clear-sky assumption on incident irradiance. The CFs presented in this work will allow users to both account for non-clear-sky conditions when using reference incident irradiance spectra and decide whether it is important for them to do so.

## Supporting Information Available

Details pertaining to the reference spectra modeled using the SMARTS tool, R packages used in this work, conversion of energy PAR to quantum PAR, maps and ridgeline plots of CFs for each month of the year, monthly and seasonal 90% confidence intervals, details of the determination of CFs for global sub-regions, monthly maps of CF standard deviation, input parameters to the direct photolysis model of triclosan in lake Greifensee, and results of modeling triclosan behavior in Moberly Lake (PDF)
Seasonal average CFs (XLSX)
Seasonal average CFs (TIF)
