# Supplementary material for: Global Corrections to Reference Irradiance Spectra for Non-Clear-Sky Conditions
Source: Environ Sci Technol. 2023 Feb 3;57(6):2682–90. doi: 10.1021/acs.est.2c07359 (PMC9933536; doi:10.1021/acs.est.2c07359)
Supplement: Supplementary file 1 — es2c07359_si_001.pdf [file es2c07359_si_001.pdf]

Supporting Information

Global corrections to reference irradiance spectra for non-clear-sky conditions

Sarah B. Partanen† and Kristopher McNeill*†

†*Institute of Biogeochemistry and Pollutant Dynamics (IBP), Department of Environmental Systems Science, ETH Zurich, 8092 Zurich, Switzerland*

Pages: 26

Figures: 19

Tables: 11

Data sources

Table S1. Input parameters to the SMARTS tool

Parameter	Value
Latitude	-75 to 75 in 5-degree increments
Altitude	0 km
Height	0 km
Default atmosphere	Variable; see Table S2 below
Water vapour	Value prescribed by the selected reference atmosphere and altitude
Ozone abundance	Default value based on the reference atmosphere
Gaseous absorption and atmospheric pollution	Light Pollution
Carbon dioxide columnar volumetric concentration	407 ppmv[1]
Extraterrestrial spectrum	Gueymard, 2004[2]
Aerosol model	Shettle and Fenn Rural[3]
Aerosol optical depth at 500 nm	0.1
Zonal albedo	None
Spectral range	400 nm – 700 nm
Solar constant	1367 W m^{-2}

Table S2. Default atmospheres used for each latitude run in the SMARTS tool

Latitude*	Default Atmosphere**
-75, -70, 70, 75	Arctic Summer; Arctic Winter
55, 60, 65	Sub-Arctic Summer; Sub-Arctic Winter
-65, -60, -55, -50, -45, -40, 40, 45, 50	Mid-Latitude Summer; Mid-Latitude Winter
-35, -30, -25, 25, 30, 35	Sub-Tropical Summer; Sub-Tropical Winter
-20, -15, -10, -5, 0, 5, 10, 15, 20	Tropical

* Note that negative latitudes represent locations south of the equator
** Note that "summer" designated default atmospheres were run for the months of March, April, May, June, July, and August for northern hemisphere latitudes, and January, February, September, October, November, and December for southern hemisphere latitudes. Conversely, "winter" designated default atmospheres were run for the months of January, February, September, October, November, and December for northern hemisphere latitudes, and March, April, May, June, July, and August for southern hemisphere latitudes.

Impact of reference atmosphere on SMARTS data

The SMARTS tool provides the option to choose from ten different reference atmospheres that can be used to simulate various atmospheric conditions. The SMARTS tool user manual notes that best results are obtained on a site-specific basis where a non-reference atmosphere is defined. However, for the purposes of this study which is looking at the whole surface of the Earth, using reference atmospheres is sufficient. The reference atmospheres were originally developed by Anderson et al.[4] and from the U.S.

Standard Atmosphere Supplements, 1966,[5] by Gueymard[6], and were generated on the basis of northern latitudes. A sensitivity analysis was performed to examine the impact of reference atmosphere on modelled SMARTS PAR data in the northern and southern hemispheres. While this does not eliminate the issue of reference atmospheres not being specifically developed for southern hemisphere latitudes, it does put into context the magnitude of the impact of reference atmosphere on the resulting SMARTS-modelled PAR values. The results of this analysis can be seen in Figure S1. The largest difference between SMARTS values modelled with the default reference atmosphere for that latitude and those modelled with an incorrect reference atmosphere is at 70 °N and °S. For April, August, September, October, and November, using the Tropical spectrum instead of the Arctic Summer or Winter spectra when modelling PAR values at 70 °S causes differences in the modelled PAR values of between 10 and 13%. The same goes for March, April, August, September, and October when modelling PAR values at 70 °N. The difference in PAR values with the use of all other reference spectra is under 10%.

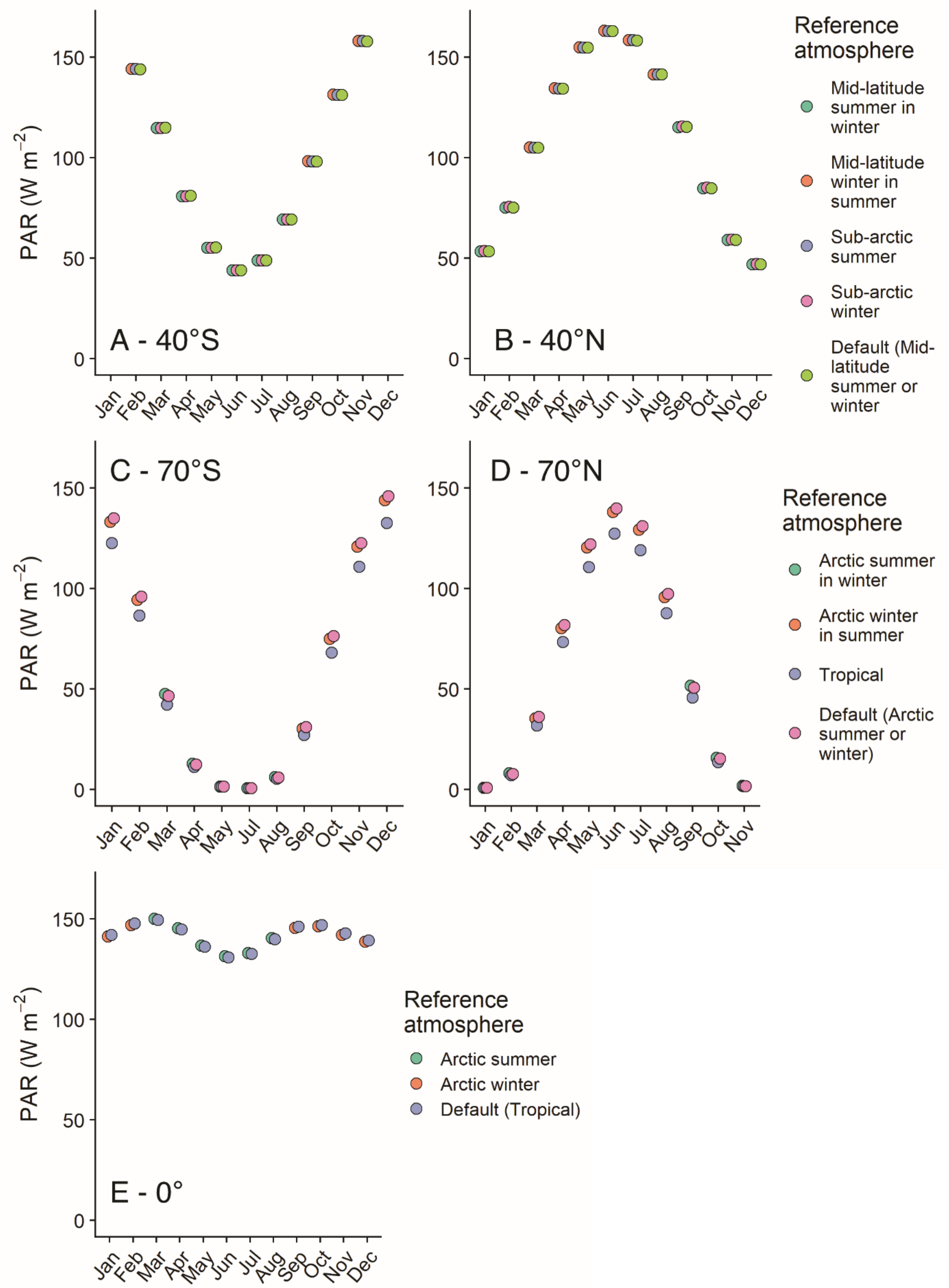

Figure S1. Results of sensitivity analysis changing the reference atmosphere used in the SMARTS model. Points labelled "summer in winter" or "winter in summer" refer to instances where the summer reference spectrum was used for winter months, or vice versa.

Clear sky PAR

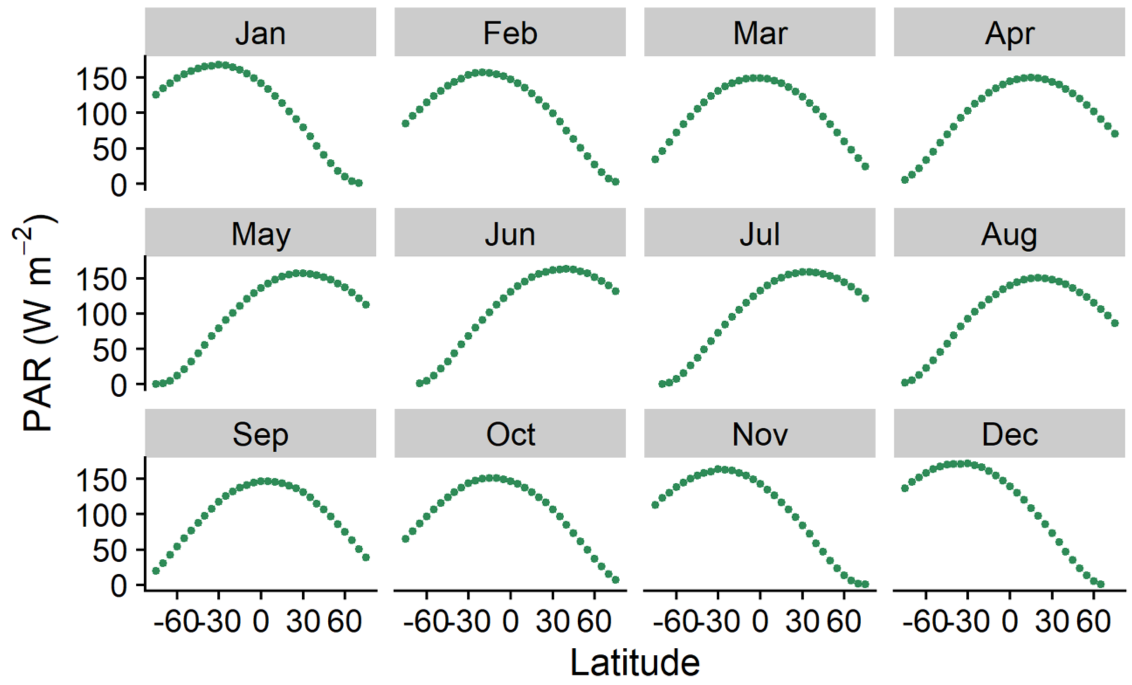

Figure S2. Monthly average SMARTS data for 2019 between 75 °S and 75 °N.

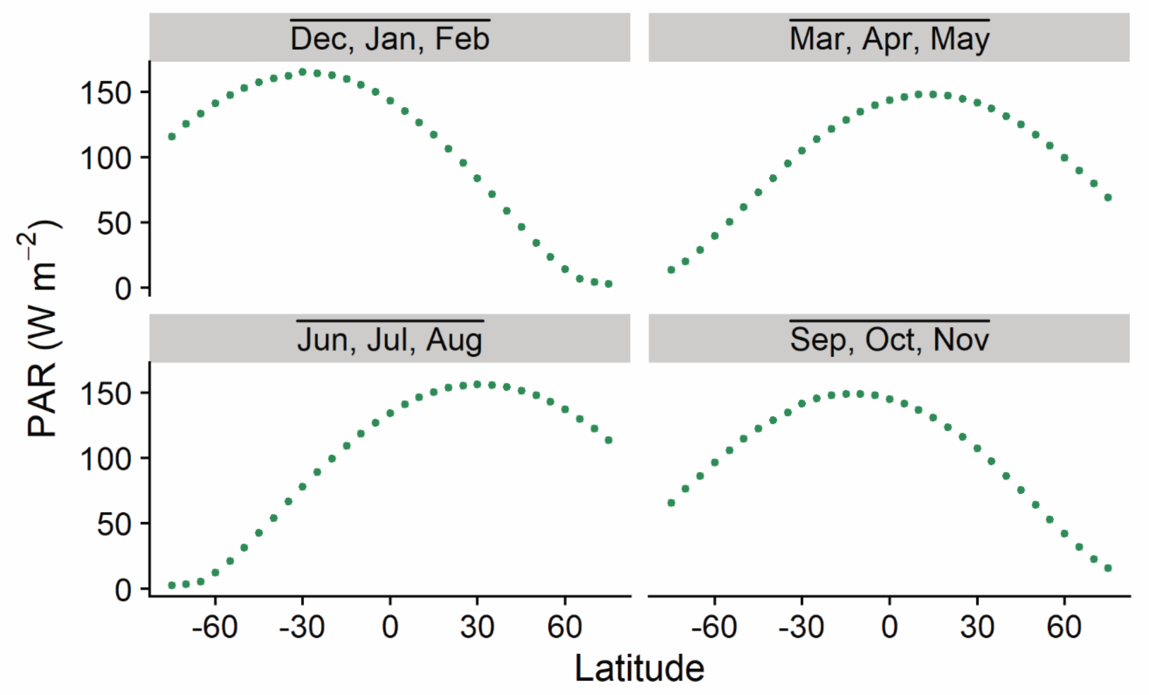

Figure S3. Seasonal average SMARTS data for 2019 between 75 °S and 75 °N.

Interpolated clear sky PAR

Data from SMARTS was modelled with a 4th order polynomial to obtain interpolated global horizontal PAR irradiance values at 1-degree latitude increments between 75 °S and 75 °N. SMARTS data was fit to Equation S1:

$$PAR = a_4 Lat^4 + a_3 Lat^3 + a_2 Lat^2 + a_1 Lat + a_0 \quad \text{(S1)}$$

Where PAR is the PAR value modelled in SMARTS (W m^{-2}), Lat is the latitude in degrees North or South, and a_4, a_3, a_2, a_1, and a_0 are fitting parameters. Equation S1 was fit to the SMARTS data modelled in 5-degree increments for each month or season for the years of 2019 (non-leap year) and 2020 (leap year). A summary of the fitting parameters is shown in Table S3, and plots showing the fitted curves are shown in Figures S4 and S5.

Table S3. Fitting parameters for polynomial models of monthly and seasonal average SMARTS data for 2019 and 2020.

					2019					2020
**Month/Season**	**a4**	**a3**	**a2**	**a1**	**a0**	**a4**	**a3**	**a2**	**a1**	**a0**
Jan	1.50e-06	1.33e-04	-2.29e-02	-1.62e+00	1.42e+02	1.51e-06	1.34e-04	-2.28e-02	-1.63e+00	1.42e+02
Feb	1.34e-06	7.57e-05	-2.62e-02	-9.93e-01	1.48e+02	1.35e-06	7.64e-05	-2.62e-02	-1.00e+00	1.48e+02
Mar	8.37e-07	1.56e-05	-2.61e-02	-1.54e-01	1.50e+02	8.36e-07	1.38e-05	-2.61e-02	-1.30e-01	1.50e+02
Apr	1.07e-06	-5.40e-05	-2.52e-02	7.53e-01	1.45e+02	1.08e-06	-5.57e-05	-2.51e-02	7.74e-01	1.45e+02
May	1.40e-06	-1.17e-04	-2.22e-02	1.44e+00	1.37e+02	1.40e-06	-1.18e-04	-2.21e-02	1.45e+00	1.36e+02
Jun	1.16e-06	-1.35e-04	-1.87e-02	1.70e+00	1.31e+02	1.16e-06	-1.35e-04	-1.87e-02	1.70e+00	1.31e+02
Jul	1.29e-06	-1.28e-04	-2.03e-02	1.57e+00	1.33e+02	1.29e-06	-1.27e-04	-2.04e-02	1.56e+00	1.33e+02
Aug	1.21e-06	-7.84e-05	-2.40e-02	1.03e+00	1.40e+02	1.20e-06	-7.68e-05	-2.41e-02	1.01e+00	1.41e+02
Sep	9.46e-07	-2.11e-05	-2.61e-02	2.47e-01	1.46e+02	9.43e-07	-1.95e-05	-2.62e-02	2.24e-01	1.46e+02
Oct	1.15e-06	4.78e-05	-2.63e-02	-6.63e-01	1.47e+02	1.16e-06	4.96e-05	-2.63e-02	-6.85e-01	1.47e+02
Nov	1.56e-06	1.18e-04	-2.42e-02	-1.44e+00	1.43e+02	1.56e-06	1.20e-04	-2.41e-02	-1.45e+00	1.43e+02
Dec	1.39e-06	1.45e-04	-2.10e-02	-1.79e+00	1.40e+02	1.40e-06	1.45e-04	-2.10e-02	-1.79e+00	1.40e+02
Dec, Jan, Feb	1.59e-06	1.26e-04	-2.39e-02	-1.48e+00	1.44e+02	1.59e-06	1.26e-04	-2.39e-02	-1.49e+00	1.44e+02
Mar, Apr, May	1.10e-06	-5.18e-05	-2.45e-02	6.78e-01	1.44e+02	1.10e-06	-5.34e-05	-2.44e-02	6.97e-01	1.44e+02
Jun, Jul, Aug	1.40e-06	-1.21e-04	-2.16e-02	1.45e+00	1.35e+02	1.40e-06	-1.21e-04	-2.17e-02	1.44e+00	1.35e+02
Sep, Oct, Nov	1.22e-06	4.83e-05	-2.56e-02	-6.19e-01	1.46e+02	1.22e-06	4.99e-05	-2.55e-02	-6.38e-01	1.46e+02

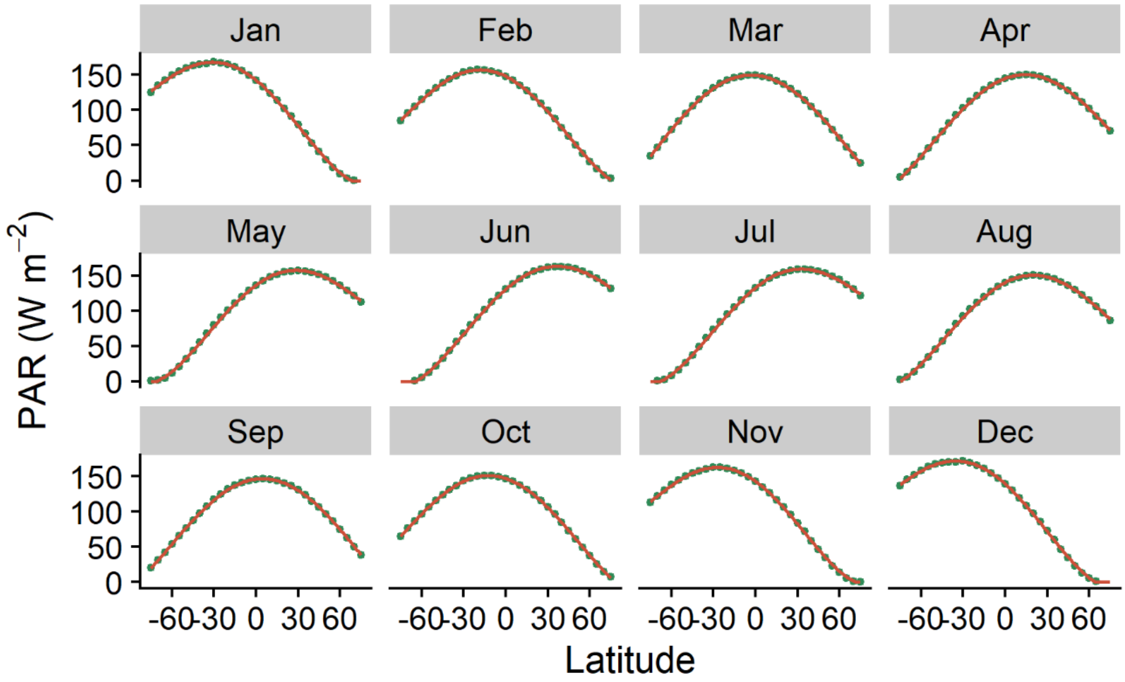

Figure S4. Polynomial fits to monthly average SMARTS data for 2019 between 75 °S and 75 °N.

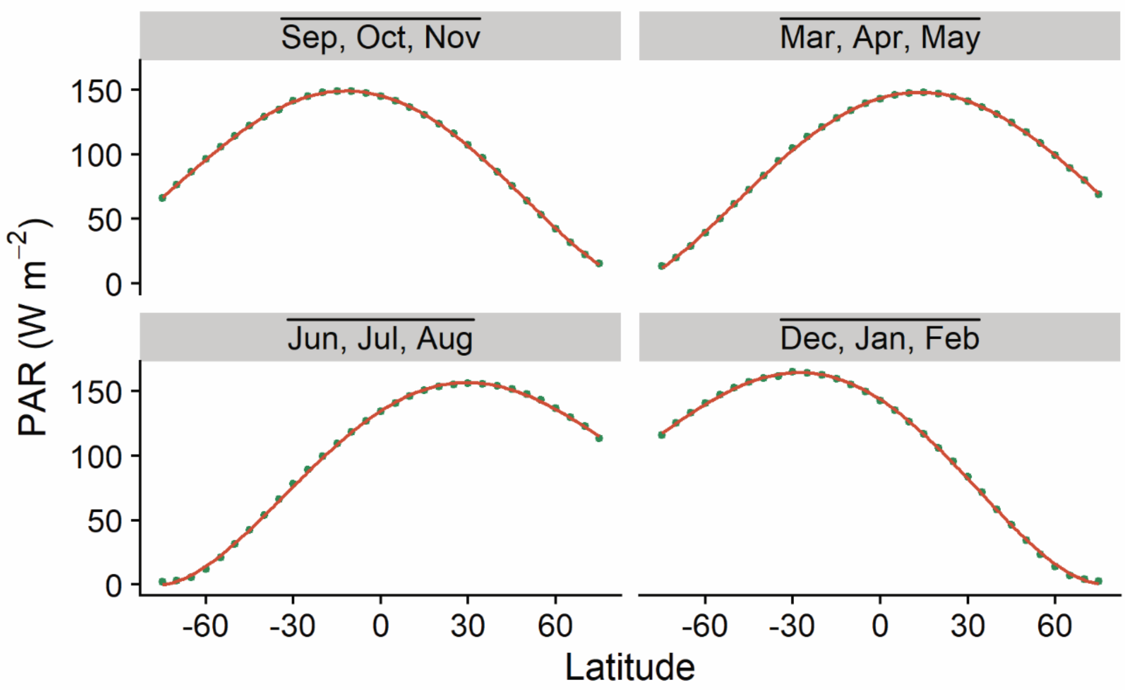

Figure S5. Polynomial fits to seasonal average SMARTS data for 2019 between 75 °S and 75 °N.

Validation PAR

Table S4. Locations and coordinates of SURFRAD stations. Directly measured PAR values at the stations were used to validate the CFs calculated in this work

Station location	Latitude (°N)	Longitude (°W)
Bondville, Illinois	40.05192	88.37309
Table Mountain, Boulder, Colorado	40.12498	105.23680
Desert Rock, Nevada	36.62373	116.10947
Fort Peck, Montana	48.30783	105.10170
Goodwin Creek, Mississippi	34.2547	89.8729
Penn. State Univ., Pennsylvania	40.72012	77.93085
Sioux Falls, South Dakota	43.73403	96.62328

Data processing and analysis

Table S5. R packages used in this work

Package name	Citation
broom	David Robinson, Alex Hayes and Simon Couch (2022). broom: Convert Statistical Objects into Tidy Tibbles. R package version 1.0.0. https://CRAN.R-project.org/package=broom
cowplot	Claus O. Wilke (2020). cowplot: Streamlined Plot Theme and Plot Annotations for 'ggplot2'. R package version 1.1.1. https://CRAN.R-project.org/package=cowplot
flextable	David Gohel (2021). flextable: Functions for Tabular Reporting. R package version 0.6.10. https://CRAN.R-project.org/package=flextable
fuzzyjoin	David Robinson (2020). fuzzyjoin: Join Tables Together on Inexact Matching. R package version 0.1.6. https://CRAN.R-project.org/package=fuzzyjoin
ggpubr	Alboukadel Kassambara (2020). ggpubr: 'ggplot2' Based Publication Ready Plots. R package version 0.4.0. https://CRAN.R-project.org/package=ggpubr
ggrepel	Kamil Slowikowski (2021). ggrepel: Automatically Position Non-Overlapping Text Labels with 'ggplot2'. R package version 0.9.1. https://CRAN.R-project.org/package=ggrepel
ggridges	Claus O. Wilke (2021). ggridges: Ridgeline Plots in 'ggplot2'. R package version 0.5.3. https://CRAN.R-project.org/package=ggridges
lubridate	Garrett Grolemund, Hadley Wickham (2011). Dates and Times Made Easy with lubridate. Journal of Statistical Software, 40(3), 1-25. URL https://www.jstatsoft.org/v40/i03/.
magrittr	Stefan Milton Bache and Hadley Wickham (2020). magrittr: A Forward-Pipe Operator for R. R package version 2.0.1. https://CRAN.R-project.org/package=magrittr
Metrics	Ben Hamner and Michael Frasco (2018). Metrics: Evaluation Metrics for Machine Learning. R package version 0.1.4. https://CRAN.R-project.org/package=Metrics
MODIStsp	L. Busetto, L. Ranghetti (2016) MODIStsp: An R package for automatic preprocessing of MODIS Land Products time series, Computers & Geosciences, Volume 97, Pages 40-48, ISSN 0098-3004, https://doi.org/10.1016/j.cageo.2016.08.020. URL https://github.com/ropensci/MODIStsp/.
raster	Robert J. Hijmans (2021). raster: Geographic Data Analysis and Modeling. R package version 3.5-2. https://CRAN.R-project.org/package=raster

RColorBrewer	Erich Neuwirth (2014). RColorBrewer: ColorBrewer Palettes. R package version 1.1-2. https://CRAN.R-project.org/package=RColorBrewer
remotes	Gábor Csárdi, Jim Hester, Hadley Wickham, Winston Chang, Martin Morgan and Dan Tenenbaum (2021). remotes: R Package Installation from Remote Repositories, Including 'GitHub'. R package version 2.4.2. https://CRAN.R-project.org/package=remotes
rgeos	Roger Bivand and Colin Rundel (2021). rgeos: Interface to Geometry Engine - Open Source ('GEOS'). R package version 0.5-8. https://CRAN.R-project.org/package=rgeos
rmapshaper	Andy Teucher and Kenton Russell (2021). rmapshaper: Client for 'mapshaper' for 'Geospatial' Operations. R package version 0.4.5. https://CRAN.R-project.org/package=rmapshaper
rnaturalearth	Andy South (2017). rnaturalearth: World Map Data from Natural Earth. R package version 0.1.0. https://CRAN.R-project.org/package=rnaturalearth
rnaturalearthdata	Andy South (2017). rnaturalearthdata: World Vector Map Data from Natural Earth Used in 'rnaturalearth'. R package version 0.1.0. https://CRAN.R-project.org/package=rnaturalearthdata
scales	Hadley Wickham and Dana Seidel (2020). scales: Scale Functions for Visualization. R package version 1.1.1. https://CRAN.R-project.org/package=scales
sf	Pebesma, E., 2018. Simple Features for R: Standardized Support for Spatial Vector Data. The R Journal 10 (1), 439-446, https://doi.org/10.32614/RJ-2018-009
skimr	Elin Waring, Michael Quinn, Amelia McNamara, Eduardo Arino de la Rubia, Hao Zhu and Shannon Ellis (2021). skimr: Compact and Flexible Summaries of Data. R package version 2.1.3. https://CRAN.R-project.org/package=skimr
terra	Robert J. Hijmans (2021). terra: Spatial Data Analysis. R package version 1.4-7. https://CRAN.R-project.org/package=terra
tidyverse	Wickham et al., (2019). Welcome to the tidyverse. Journal of Open Source Software, 4(43), 1686, https://doi.org/10.21105/joss.01686

Conversion of quantum PAR to energy PAR

Indirectly measured real sky PAR data over ocean surfaces is provided in quantum PAR units (mol photon m^{-2} s^{-1}), and was converted to energy units (W m^{-2}) using a weighted average conversion factor approach.

First, a conversion factor for each PAR wavelength (400 – 700 nm) was calculated using the following equation:

$$f_{conv,\lambda} = \frac{N_A \cdot c \cdot h}{86400 \cdot \lambda} \quad \text{(S2)}$$

where $f_{conv,\lambda}$ is the conversion factor for each PAR wavelength (W day mol^{-1}), N_A is Avogadro's number (6.02 x 10^{23} mol^{-1}), c is the speed of light (3.0 x 10^{8} m s^{-1}), h is Planck's constant (6.63 x 10^{-34} kg m^{2} s^{-1}), λ is wavelength (m), and 86400 is the conversion factor between seconds and days.

Next, a weighted average conversion factor was obtained for each day of the year at a given latitude. This was done because there are a different number of photons at

each wavelength, and so each wavelength of PAR light should not contribute equally to the conversion between photons of light and energy of light. The relative number of photons at each PAR wavelength was obtained using SMARTS spectra for a given latitude. The global horizontal photosynthetic photon flux at each wavelength was divided by the sum of the photon flux values over the PAR spectrum. Next, the weighted average conversion factor was calculated using Equation S3.

$$f_{conv,weighted} = \sum_{\lambda} \frac{f_{conv,\lambda} \cdot N_{rel,phot,\lambda}}{\sum_{\lambda} N_{rel,phot,\lambda}} \qquad \text{(S3)}$$

where $f_{conv,weighted}$ is the weighted average conversion factor from quantum PAR to energy PAR for a given day and latitude (W day mol^{-1}), and $N_{rel,phot,\lambda}$is the relative number of photons at each PAR wavelength. $f_{conv,weighted}$ was found to be insensitive to the day of the year for all latitudes. The difference between minimum and maximum $f_{conv,weighted}$ values ranged from 0.014 at 60°S to 0.001 at 0°. Therefore, $f_{conv,weighted}$ values were averaged over all days of the year to obtain a single value of $f_{conv,weighted}$ for a given latitude.

$f_{conv,weighted}$ was calculated for multiple latitudes, as shown in Table S6.

Table S6. Values of $f_{conv,weighted}$ at different latitudes

**Latitude**	$f_{conv,weighted}$
60°N	2.532
40°N,	2.536
20°N	2.540
0°	2.540
20°S	2.540
40°S	2.540
60°S	2.534

As the values of $f_{conv,weighted}$ are also insensitive to latitude, the average of the values in Table S6 was used as the conversion factor for indirectly measured real sky PAR satellite data over oceans.

To check the conversion of real sky PAR data over oceans from quantum PAR units to energy PAR units, PAR values over eight large lakes were compared, as these are areas of overlap between the two PAR datasets over land and ocean surfaces. The

lakes examined were chosen to span a wide range of latitudes and longitudes and are summarized in Table S7.

Table S7: Location and coordinates of the eight lakes used to compare land and ocean PAR values

**Lake Name**	**Location**	**Latitude**	**Longitude**
Lake Argyle	Australia	16.43 °S	128.77 °E
Lake Balkhash	Kazakhstan	45.91 °N	73.95 °E
Lake Baikal	Russia	53.63 °N	108.14 °E
Great Salt Lake	United States	41.2 °N	112.5 °W
Qinghai Lake	China	36.89 °N	100.18 °E
Lake Titicaca	Bolivia/Peru	15.92 °S	69.3 °E
Lake Tuz	Turkey	38.86 °N	33.36 °E
Lake Victoria	Burundi, Kenya, Rwanda, Tanzania, Uganda	1.3 °S	33.23 °E

Polygons of PAR data from the two datasets were extracted for the eight lakes in Table S7, and the PAR values of corresponding pixels were divided to ascertain the differences between the converted indirectly measured real sky PAR data over oceans and the indirectly measured real sky PAR data over land. The ratio of converted ocean PAR to land PAR was averaged over all pixels of data for each lake for every month of the year. A summary of this analysis can be seen in Figure S6.

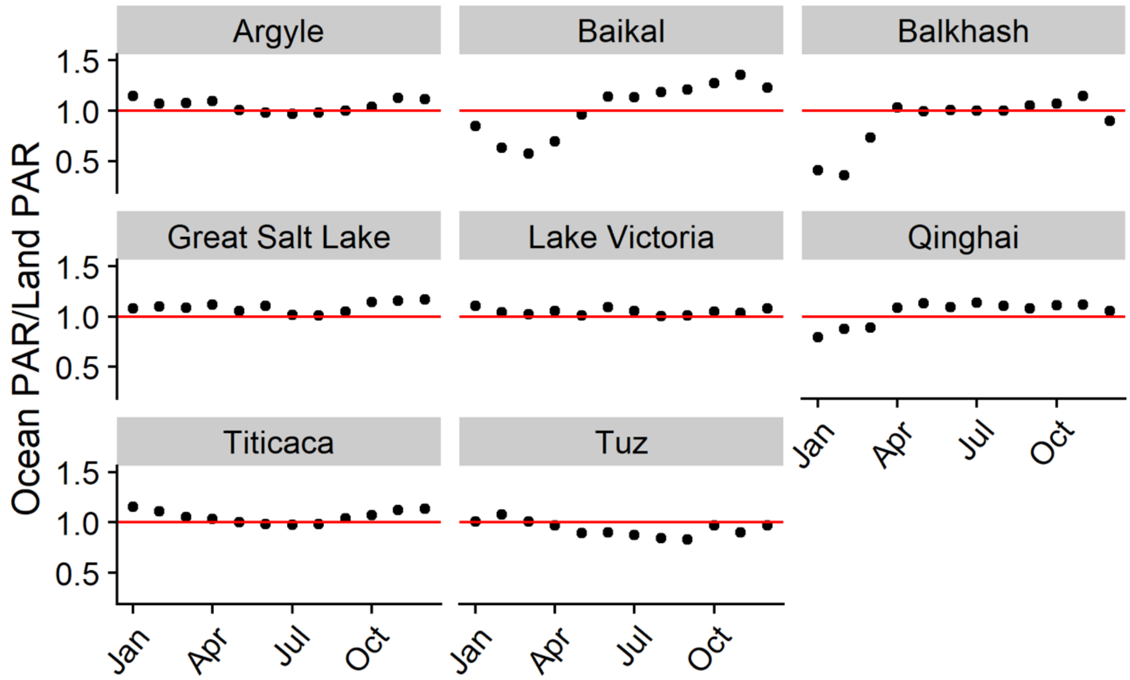

Figure S6. Comparison of indirectly measured real sky PAR values over oceans and over land for eight lakes. PAR values over oceans were converted from quantum PAR to energy PAR units using the process described above.

In Figure S6, the closer a value is to 1 the more similar are the converted ocean PAR values to the land PAR values. PAR values over Lake Baikal are the most dissimilar, as are those for the months of January to March for Lake Balkhash. In this work we prioritized the indirectly measured PAR data over land for areas of overlap.

Results and discussion

Global correction factors

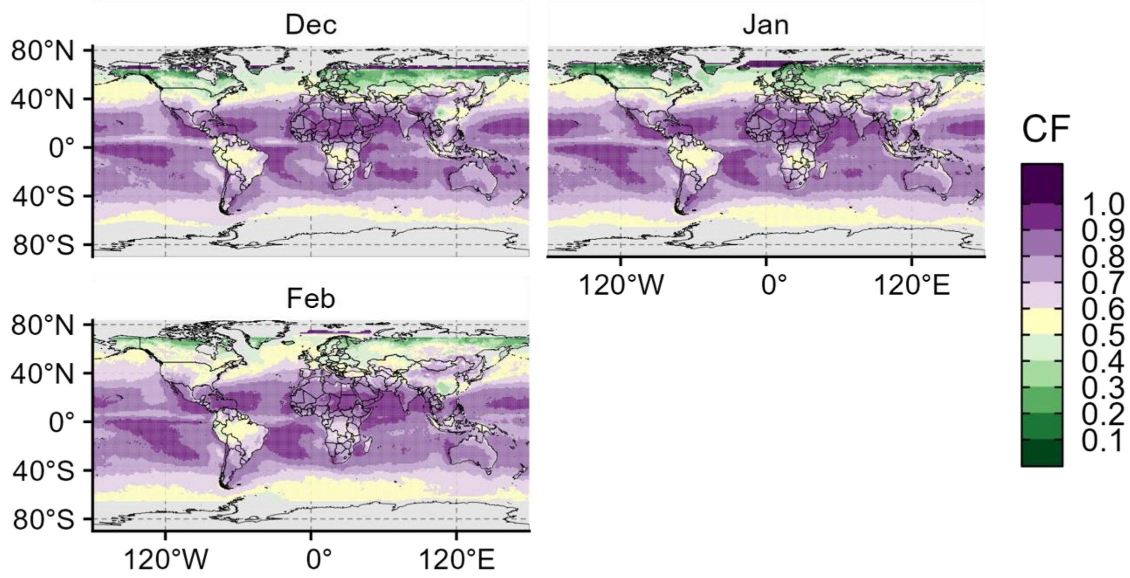

Figure S7: Global maps of CFs for the months of December, January, and February

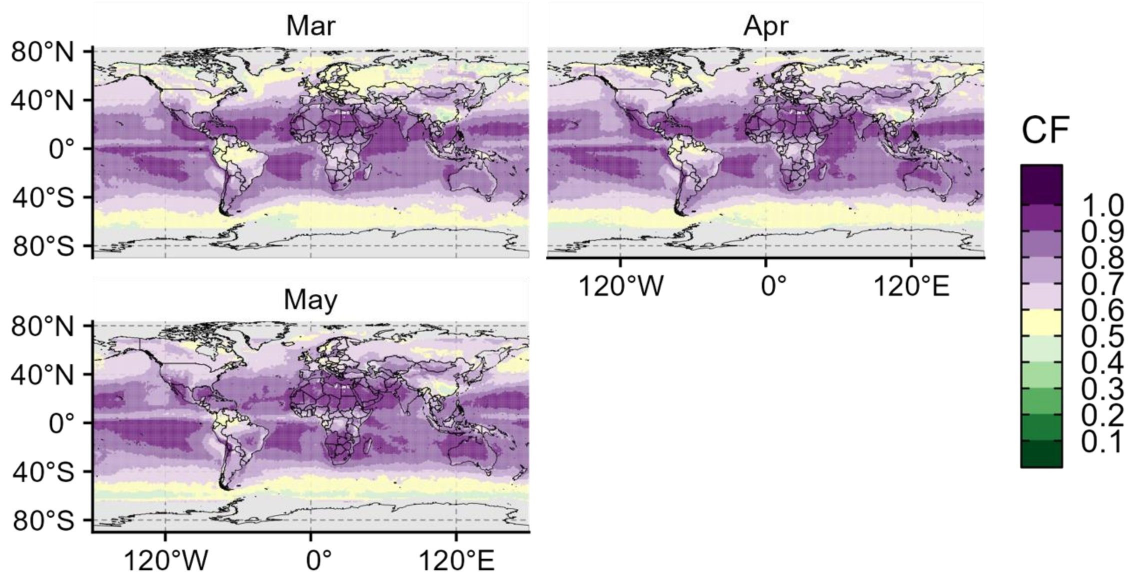

Figure S8: Global maps of CFs for the months of March, April, and May

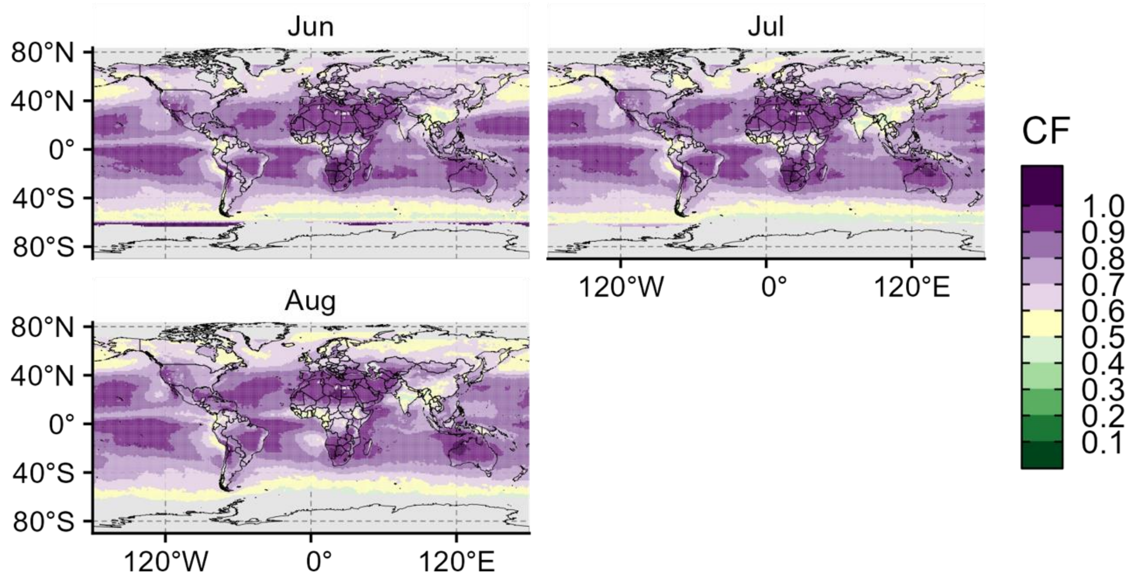

Figure S9: Global maps of CFs for the months of June, July, and August

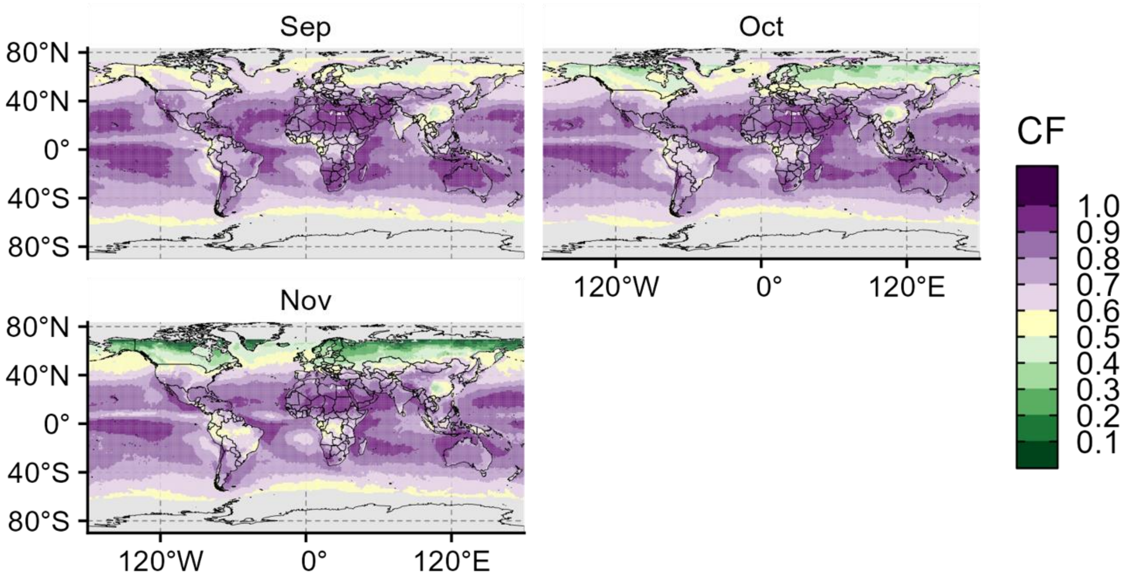

Figure S10: Global maps of CFs for the months of September, October, and November

Table S8: 90% confidence interval for seasonal CFs spatially averaged across all latitudes

	**90% confidence interval**	
**Season**	**Lower limit**	**Upper limit**
$\overline{D,J,F}$	0.38	0.92
$\overline{M,A,M}$	0.54	0.93
$\overline{J,J,A}$	0.52	0.95
$\overline{S,O,N}$	0.49	0.93

Table S9: 90% confidence interval for monthly CFs spatially averaged across all latitudes

	**90% confidence interval**	
**Month**	**Lower limit**	**Upper limit**
January	0.35	0.93
February	0.45	0.93
March	0.52	0.93
April	0.53	0.93
May	0.52	0.94
June	0.53	0.95
July	0.52	0.95
August	0.54	0.95
September	0.54	0.94
October	0.44	0.93
November	0.33	0.93
December	0.37	0.93

Variability of correction factors

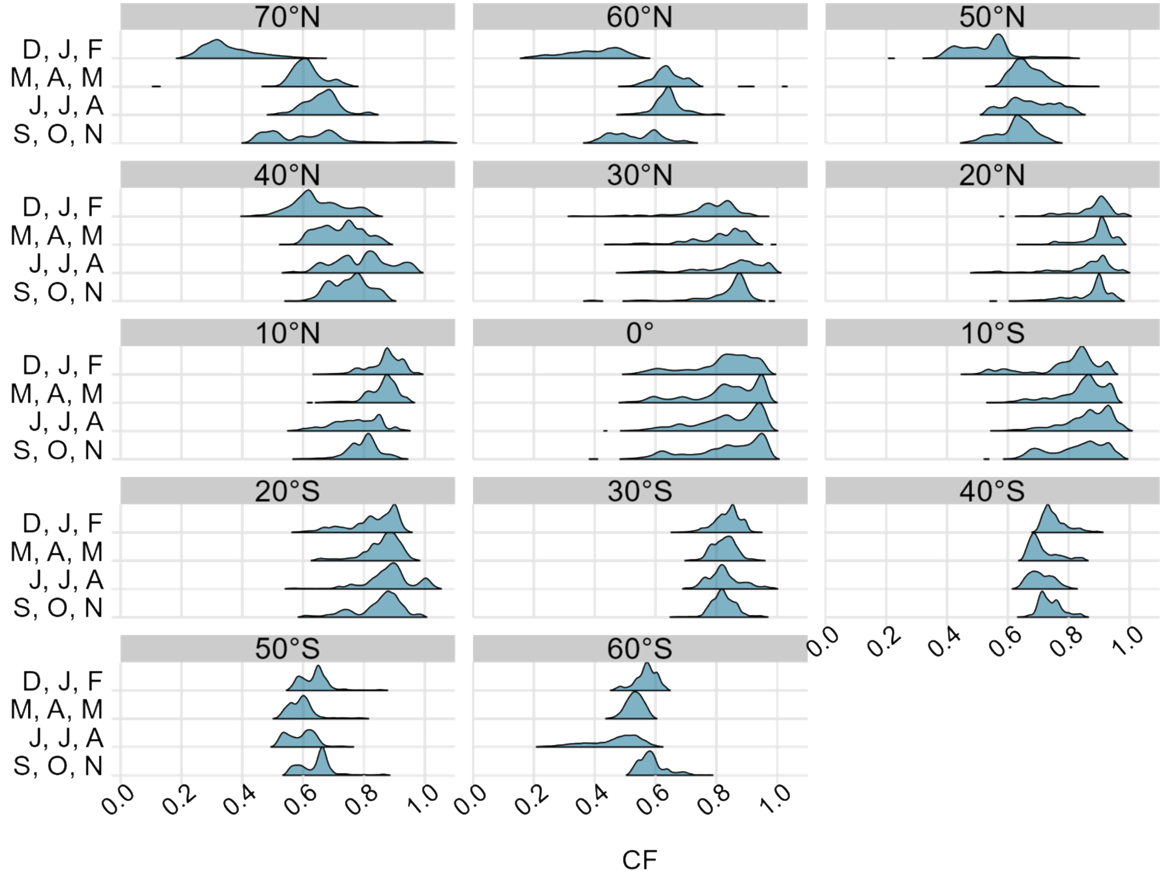

Figure S11. Ridgeline plots of CFs for latitude bands in 10° increments broken out by season

Table S10. Countries belonging to each sub-region of the world

Region	Subregion				
	East Africa	Mid Africa	North Africa	South Africa	West Africa
Africa	Burundi	Angola	Algeria	Botswana	Benin
	Djibouti	Central African Republic	Egypt	Lesotho	Burkina Faso
	Eritrea	Cameroon	Libya	Namibia	Ivory Coast
	Ethiopia	Democratic Republic of the Congo	Morocco	Swaziland	Ghana
	Kenya	Republic of Congo	Western Sahara	South Africa	Guinea
	Madagascar	Gabon	Sudan		Gambia
	Mozambique	Equatorial Guinea	Tunisia		Guinea-Bissau
	Malawi	Chad			Liberia
	Rwanda				Mali
	South Sudan				Mauritania
	Somaliland				Niger
	Somalia				Nigeria
	Tanzania				Senegal

	Uganda				Sierra Leone
	Zambia				Togo
	Zimbabwe				
	**Caribbean**	**Central America**	**North America**	**South America**	
**Americas**	The Bahamas	Belize	Canada	Argentina	
	Cuba	Costa Rica	Greenland	Bolivia	
	Dominican Republic	Guatemala	United States of America	Brazil	
	Haiti	Honduras		Chile	
	Jamaica	Mexico		Colombia	
	Puerto Rico	Nicaragua		Ecuador	
	Trinidad and Tobago	Panama		Falkland Islands	
		El Salvador		Guyana	
				Peru	
				Paraguay	
				Suriname	
				Uruguay	
				Venezuela	
	**Central Asia**	**East Asia**	**South Asia**	**Southeast Asia**	**West Asia**
**Asia**	Kazakhstan	China	Afghanistan	Brunei	United Arab Emirates
	Kyrgyzstan	Japan	Bangladesh	Indonesia	Armenia
	Tajikistan	South Korea	Bhutan	Cambodia	Azerbaijan
	Turkmenistan	Mongolia	India	Laos	Northern Cyprus
	Uzbekistan	North Korea	Iran	Myanmar	Cyprus
		Taiwan	Sri Lanka	Malaysia	Georgia
			Nepal	Philippines	Iraq
			Pakistan	Thailand	Israel
				East Timor	Jordan
				Vietnam	Kuwait
					Lebanon
					Oman
					Palestine
					Qatar
					Saudi Arabia
					Syria
					Turkey
					Yemen
	**East Europe**	**North Europe**	**South Europe**	**West Europe**	
**Europe**	Bulgaria	Denmark	Albania	Austria	
	Belarus	Estonia	Bosnia and Herzegovina	Belgium	

	Czech Republic	Finland	Spain	Switzerland	
	Hungary	United Kingdom	Greece	Germany	
	Moldova	Ireland	Croatia	France	
	Poland	Iceland	Italy	Luxembourg	
	Romania	Lithuania	Kosovo	Netherlands	
	Russia	Latvia	Macedonia		
	Slovakia	Norway	Montenegro		
	Ukraine	Sweden	Portugal		
			Serbia		
			Slovenia		
	**Australia and New Zealand**	**Melanesia**			
**Oceania**	Australia	Fiji			
	New Zealand	New Caledonia			
		Papua New Guinea			
		Solomon Islands			
		Vanuatu			

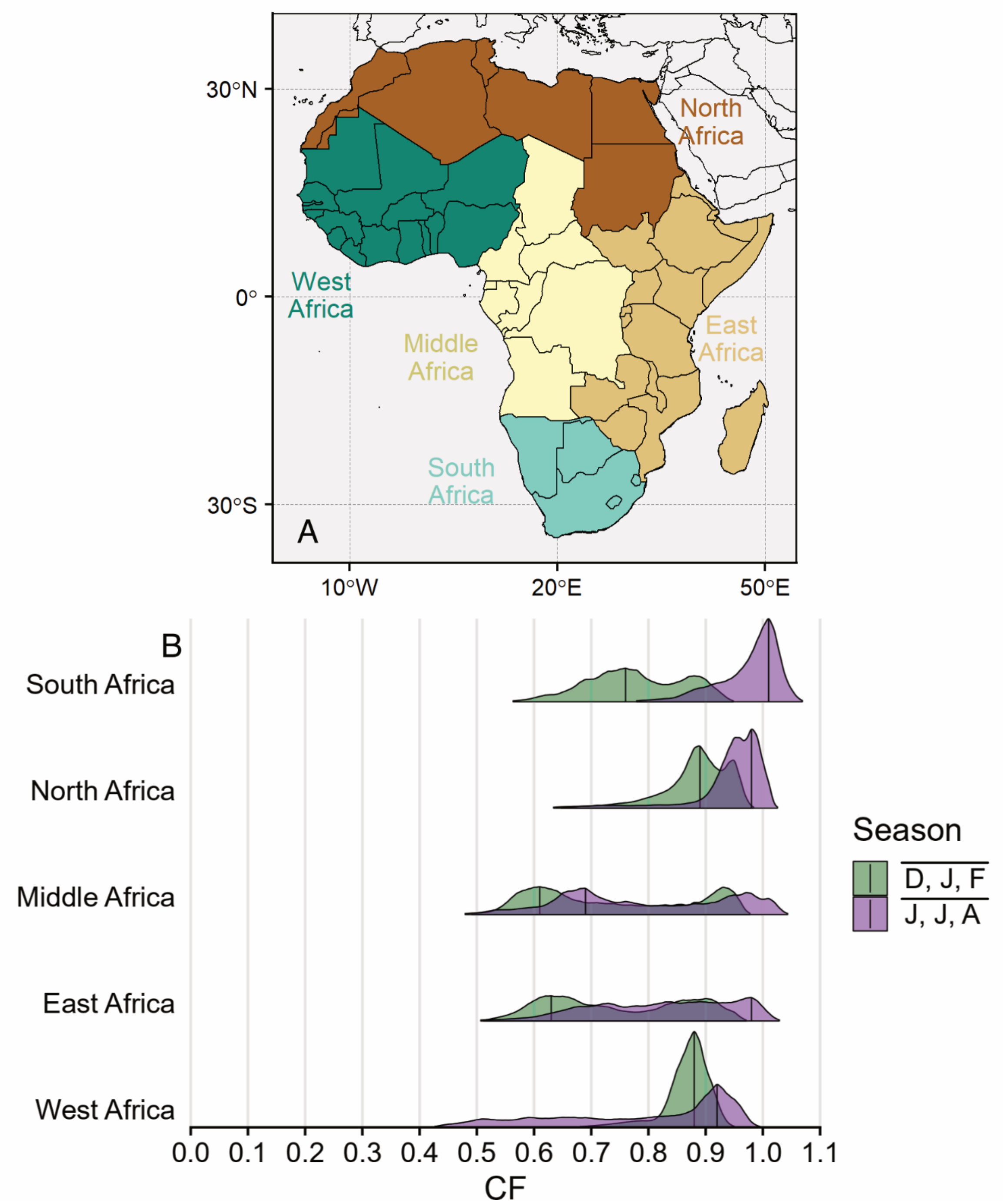

Figure S12. A: Map defining the subregions in Africa. B: Ridgeline plots of CFs for the seasons December, January, and February ($\overline{D, J, F}$) and June, July, and August ($\overline{J, J, A}$) for the subregions in Africa. The vertical lines in each density distribution represent the mode of the distribution.

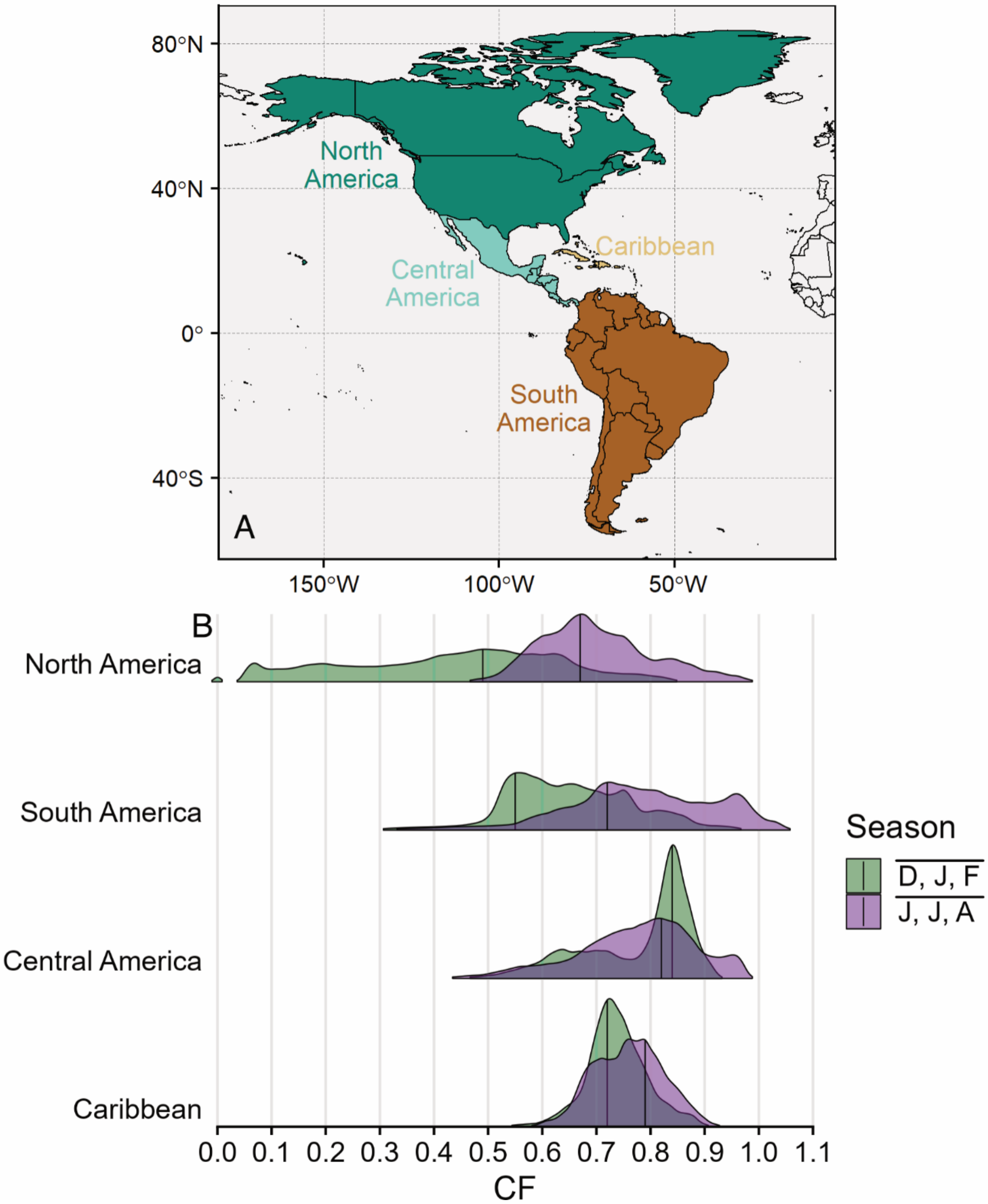

Figure S13. A: Map defining the subregions in the Americas. B: Ridgeline plots of CFs for the seasons December, January, and February ($\overline{D,J,F}$) and June, July, and August ($\overline{J,J,A}$) for the subregions in the Americas. The vertical lines in each density distribution represent the mode of the distribution.

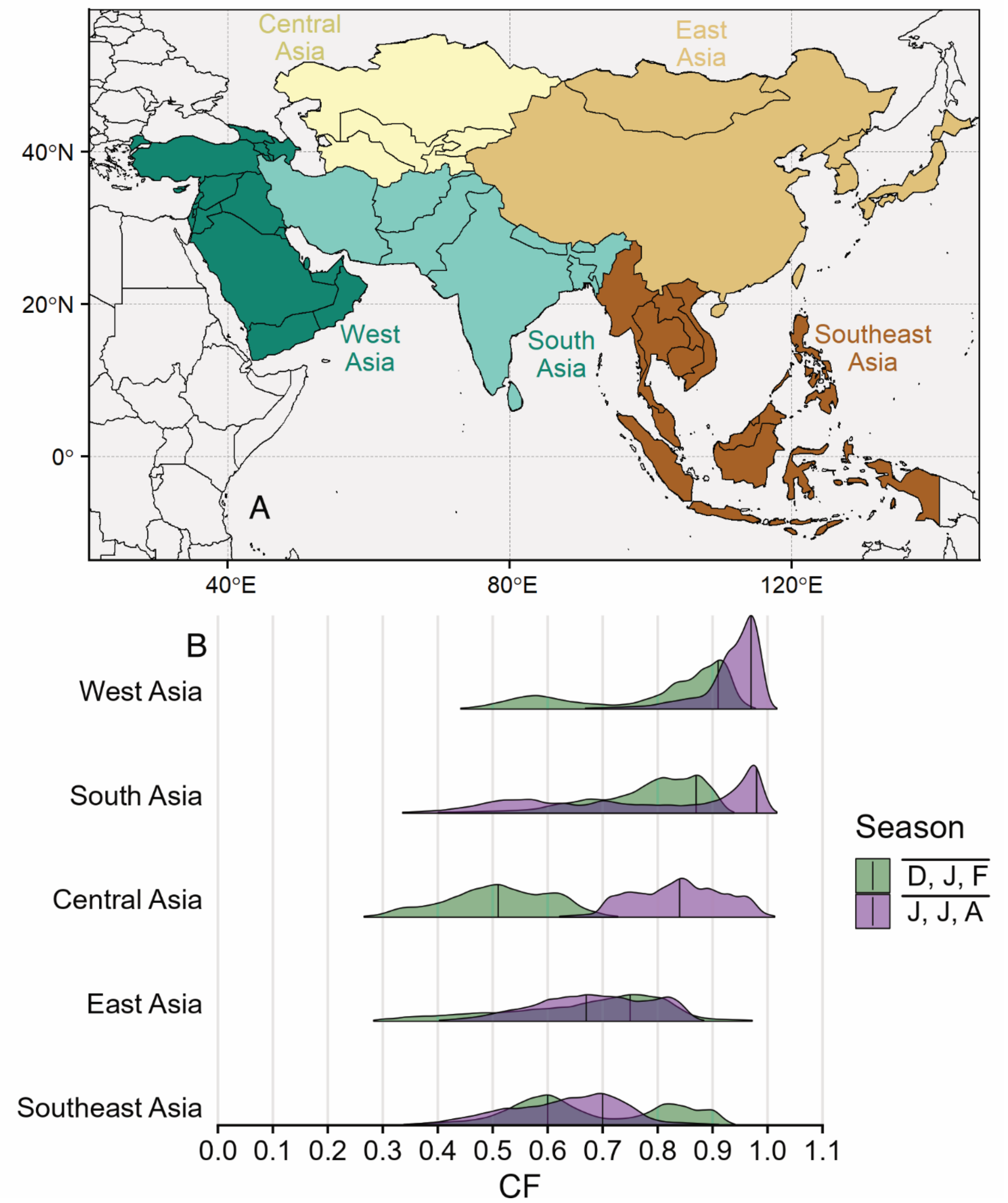

Figure S14. A: Map defining the subregions in Asia. B: Ridgeline plots of CFs for the seasons December, January, and February ($\overline{D, J, F}$) and June, July, and August ($\overline{J, J, A}$) for the subregions in Asia. The vertical lines in each density distribution represent the mode of the distribution.

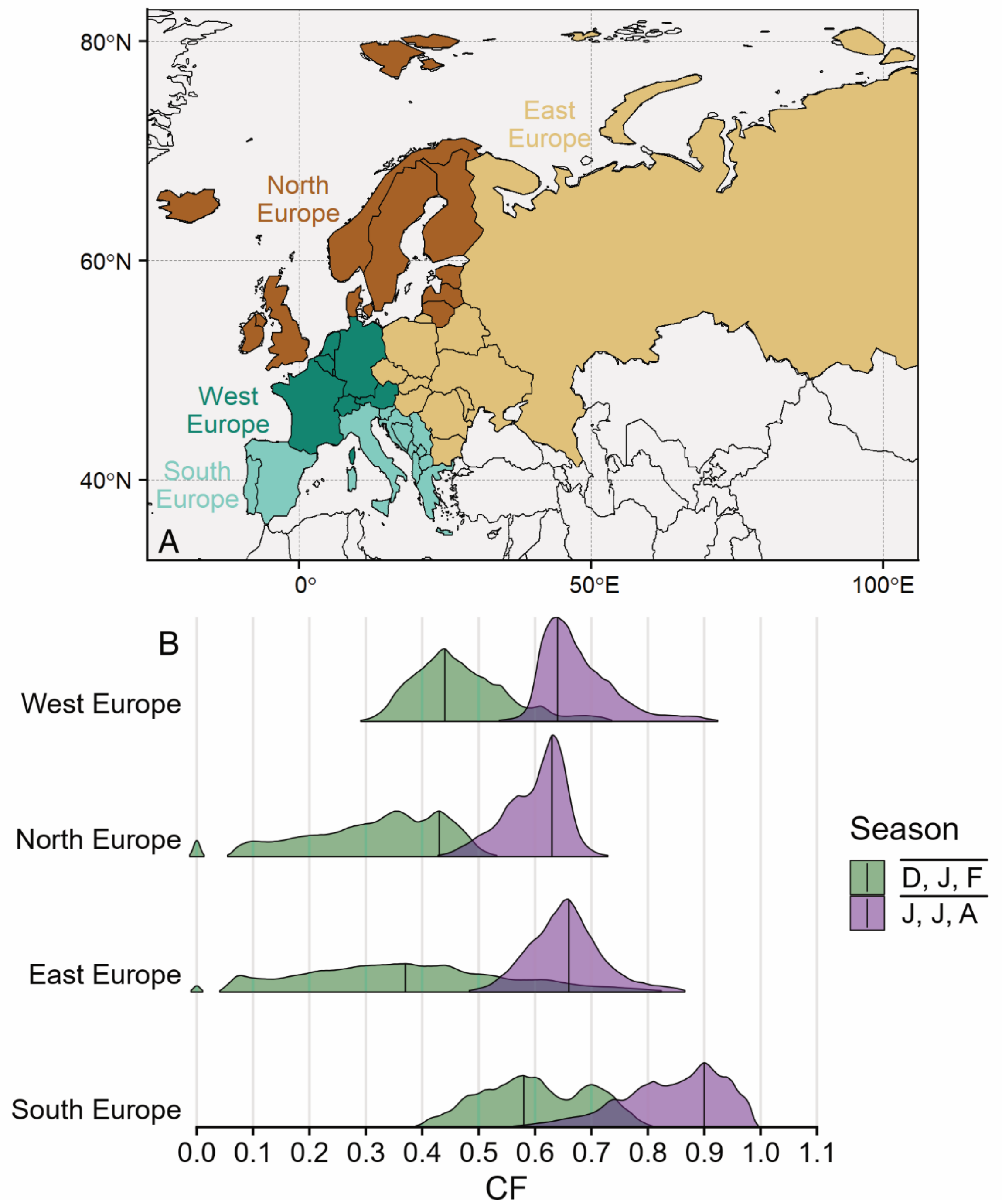

Figure S15. A: Map defining the subregions in Europe. B: Ridgeline plots of CFs for the seasons December, January, and February ($\overline{D,J,F}$) and June, July, and August ($\overline{J,J,A}$) for the subregions in Europe. The vertical lines in each density distribution represent the mode of the distribution.

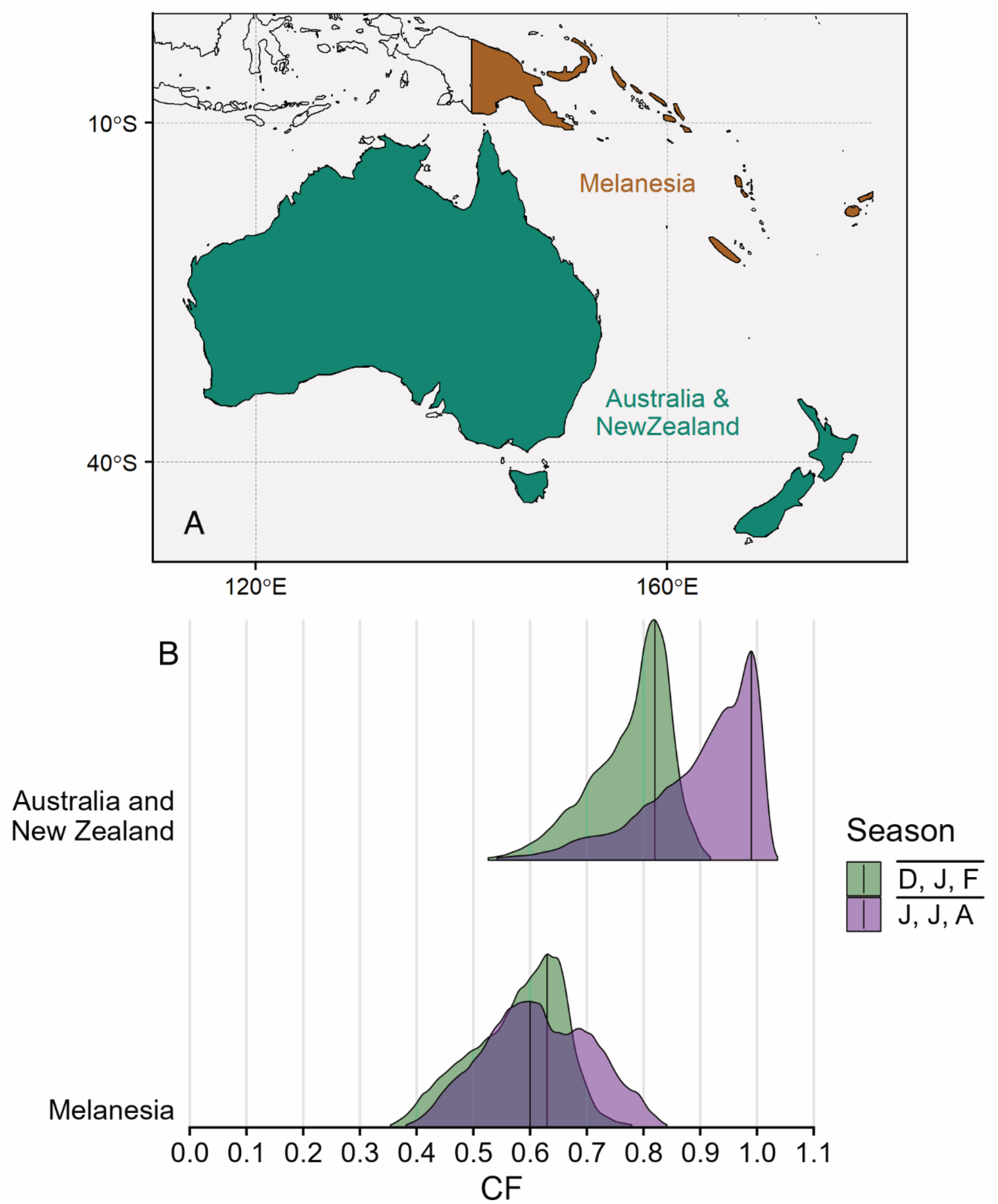

Figure S16. A: Map defining the subregions in Oceania. B: Ridgeline plots of CFs for the seasons December, January, and February $(\overline{D,J,F})$ and June, July, and August $(\overline{J,J,A})$ for the subregions in Oceania. The vertical lines in each density distribution represent the mode of the distribution.

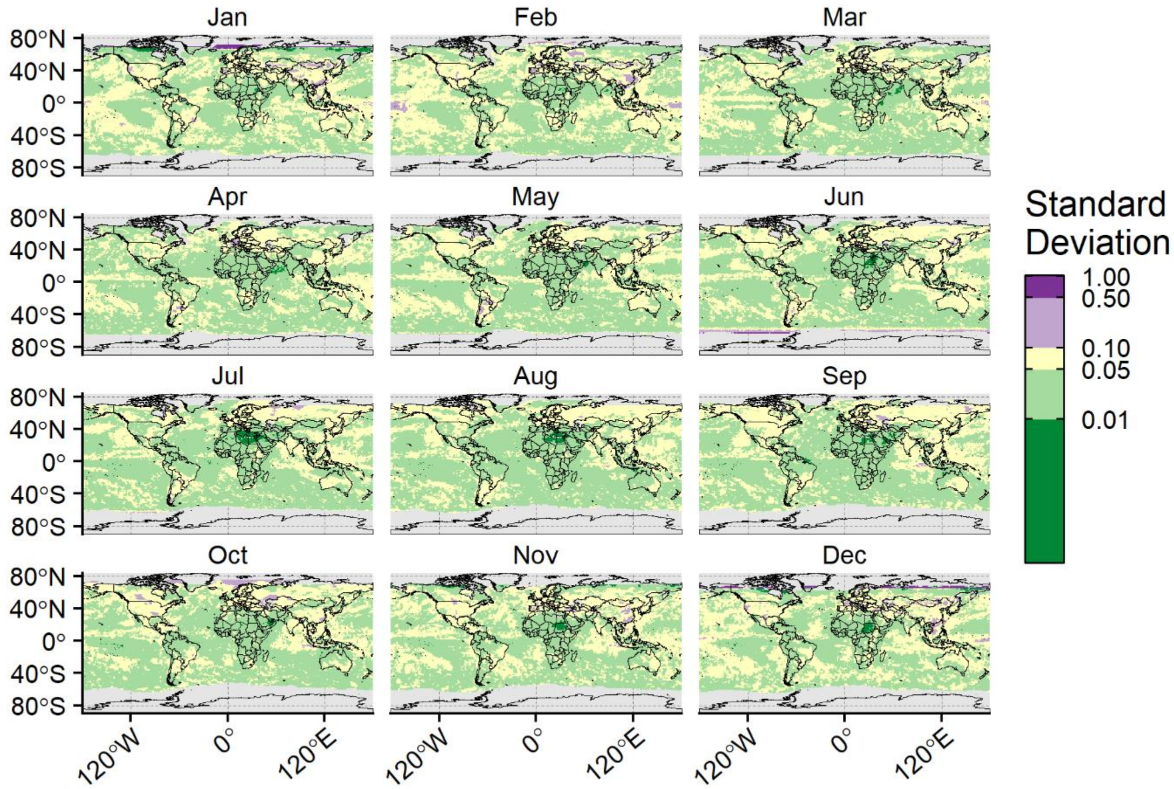

Figure S17. Monthly maps of standard deviation in CF

Triclosan in lake Greifensee

The rate constant for the direct photolysis of triclosan was calculated according to equation 2 in the main text. Figure S18 shows the diffuse attenuation coefficient for Lake Greifensee and the molar absorptivity for triclosan in the phenolate form, both used as wavelength-dependent input parameters to equation 2 in the main text.

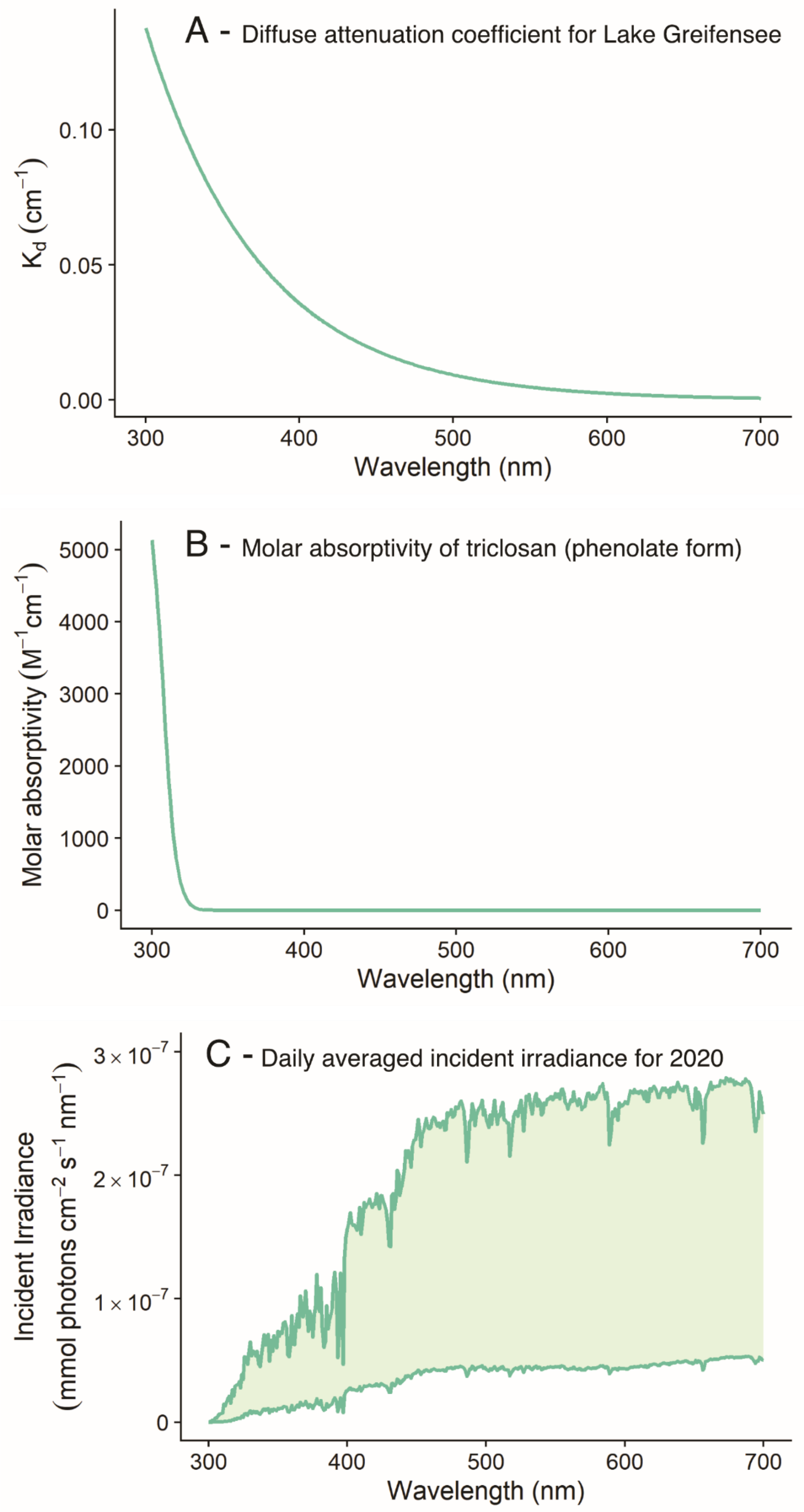

Figure S18. Input parameters for modelling k_{TCS}^{dir}

Table S11. Comparison using a Student's t-test of the means of the direct photolysis rate constant of triclosan in Lake Greifensee calculated using clear sky PAR or clear sky PAR multiplied by the CF. $p > 0.05$ = ns, $p \leq 0.05$ = *, $p \leq 0.01$ = **, $p \leq 0.001$ = ***, $p \leq 0.0001$ = ****

Season	p-value	Significance level
Winter	1.9×10^{-9}	****
Spring	1.9×10^{-5}	****
Summer	2.1×10^{-12}	****
Autumn	1.5×10^{-4}	***

Fate of triclosan in Moberly Lake

Moberly Lake is an oligotrophic lake in northern British Columbia. Triclosan fate in Moberly Lake was modelled in the same way as it was for lake Greifensee, but using lake-specific parameters for mixing patterns, clear-sky irradiance, CFs, and mean lake depth. The DOC in Moberly Lake was not known, and so was assumed to be 4 mg/L, the same as lake Greifensee. This is in line with global-scale predictions of lake DOC.[7] The epilimnion depth of Moberly Lake was also not known, and so was modelled according to Hanna.[8]

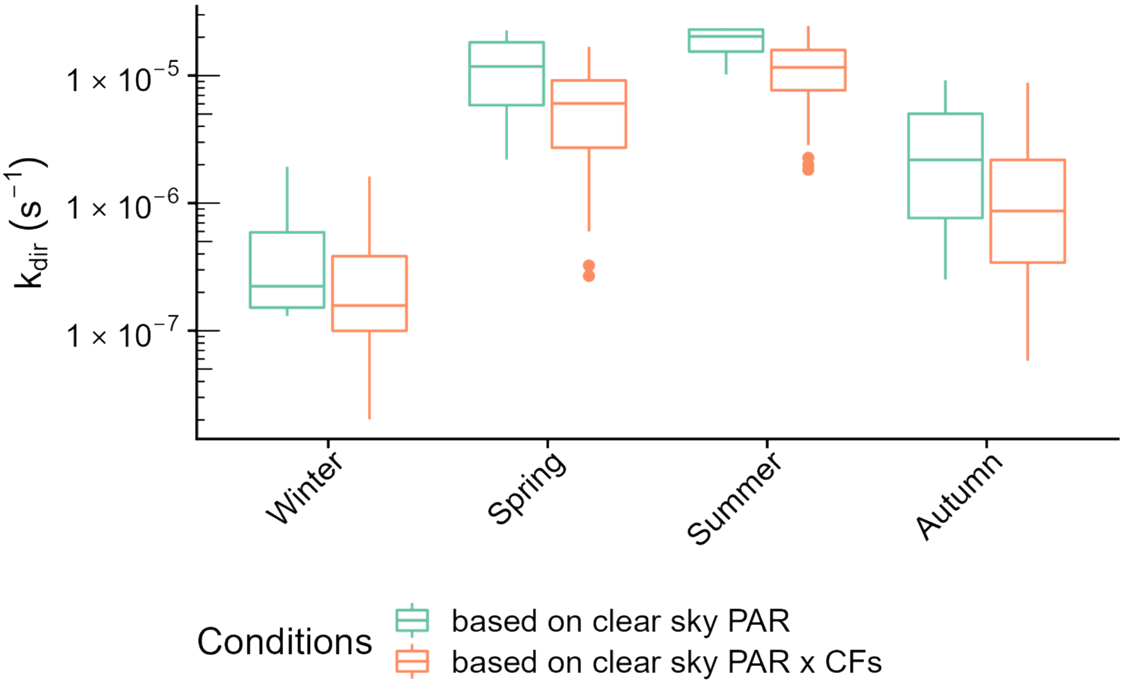

Figure S19. Range of seasonal direct photolysis rate constants of triclosan at the surface of Moberly Lake. Green values were calculated using clear sky PAR, and orange values were calculated using clear sky PAR multiplied by the CFs generated in this work. Note that the y-axis is log-scale.

References

(1) Apell, J. N.; McNeill, K. Updated and Validated Solar Irradiance Reference Spectra for Estimating Environmental Photodegradation Rates. *Environ. Sci. Process. Impacts* **2019**, *21* (3), 427–437. https://doi.org/10.1039/C8EM00478A.

(2) Gueymard, C. A. The Sun's Total and Spectral Irradiance for Solar Energy Applications and Solar Radiation Models. *Sol. Energy* **2004**, *76* (4), 423–453. https://doi.org/10.1016/j.solener.2003.08.039.

(3) Shettle, E.; Fenn, R. Models for the Aerosols of the Lower Atmosphere and the Effects of Humidity Variations on Their Optical Properties. *Env. Res* **1979**, 94.

(4) Anderson, G. P.; Clough, S. A.; Kneizys, F. X.; Chetwynd, J. H.; Shettle, E. P. *AFGL Atmospheric Constituent Profiles (0.120km)*; AIR FORCE GEOPHYSICS LAB HANSCOM AFB MA, 1986.

(5) *U.S. Standard Atmosphere Supplements, 1966*; NASA-CR-88870; 1966.

(6) Gueymard, C. *Simple Model for the Atmospheric Radiative Transfer of Sunshine (SMARTS2) Algorithms and Performance Assessment*; FSEC-PF-270-95; Florida Solar Energy Center: Cocoa, FL, 1995; p 84.

(7) Toming, K.; Kotta, J.; Uuemaa, E.; Sobek, S.; Kutser, T.; Tranvik, L. J. Predicting Lake Dissolved Organic Carbon at a Global Scale. *Sci. Rep.* **2020**, *10*, 8471. https://doi.org/10.1038/s41598-020-65010-3.

(8) Hanna, M. Evaluation of Models Predicting Mixing Depth. *Can. J. Fish. Aquat. Sci.* **1990**, *47* (5), 940–947. https://doi.org/10.1139/f90-108.
